# Chemical Feedstock Recovery Through Plastic Pyrolysis: Challenges and Perspectives Toward a Circular Economy

**DOI:** 10.1002/cssc.202500210

**Published:** 2025-06-10

**Authors:** Shogo Kumagai, Kazuki Fujiwara, Toru Nishiyama, Yuko Saito, Toshiaki Yoshioka

**Affiliations:** ^1^ Graduate School of Environmental Studies Tohoku University 6‐6‐07 Aoba, Aramaki‐aza, Aoba‐ku Sendai 980‐8579 Japan; ^2^ Graduate School of Engineering Tohoku University 6‐6‐07 Aoba, Aramaki‐aza, Aoba‐ku Sendai 980‐8579 Japan; ^3^ Environmental Solution Department DOWA ECO‐SYSTEM CO. LTD. 14‐1, Sotokanda 4‐Chome Chiyoda‐ku Tokyo 101‐0021 Japan

**Keywords:** circular economy, feedstock recycling, liquefaction, plastics, pyrolysis

## Abstract

Plastics are indispensable in daily life, with both production and waste generation increasing annually. As the world strives for net‐zero emissions, advancing plastic recycling technologies has become a global priority. Pyrolytic liquefaction is a promising approach for recovering chemical feedstocks, including fuel fractions, from waste plastics, potentially substituting petroleum resources. Since the 1970s, research on pyrolytic liquefaction has progressed globally, and several industrial‐scale plants are now in operation. However, to accelerate the transition to a circular economy, it is crucial to bridge the knowledge gap between lab‐scale research and industrial‐scale implementation of pyrolysis‐liquefaction technologies. This review provides a comprehensive analysis of the current state of plastic recycling, the progress and challenges in cutting‐edge lab‐scale research on pyrolytic liquefaction, alongside the latest trends in industrial‐scale liquefaction projects. It reveals that pyrolytic liquefaction of a wide range of plastics—including halogenated plastics and poly(ethylene terephthalate)—has been extensively studied at the laboratory level. In contrast, industrial‐scale operations often focus on more common, easily pyrolyzed plastics and generally avoid the use of catalysts. This highlights the urgent need to develop robust, reusable, and cost‐effective catalysts, as well as optimized process designs, to expand the range of plastic feedstocks suitable for industrial‐scale pyrolysis plants.

## Graphical abstract

This review examines the progress and challenges of lab‐scale pyrolytic liquefaction, trends in industrial‐scale projects, and the gap between these technologies, proposing a future research direction for plastic pyrolysis to advance the transition to a circular economy.

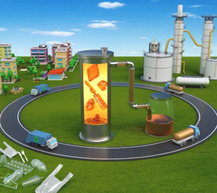

## 1 Introduction

The transition to a circular economy has become a key focus in global environmental trend. The Ellen Macarthur Foundation's 2013 report, “Towards a Circular Economy,”^[^ ^1^ ^]^ garnered widespread attention. The European Union (EU), which is committed to a wide range of environmental policies, also launched a series of policies in this area, including the first version of its “Circular Economy Action Plan”^[^ ^2^ ^]^ and a revised version.^[^ ^3^ ^]^ According to the EU, “circular economy” means an economic system whereby the value of products, materials, and other resources in the economy is maintained for as long as possible, enhancing their efficient use in production and consumption, thereby reducing the environmental impact of their use, minimizing waste and the release of hazardous substances at all stages of their life cycle, including through the application of the waste hierarchy.^[^ ^4^ ^]^ Therefore, efficient recycling is necessary to achieve the transition.

It has also been emphasized that plastics require particular attention in the transition to a circular economy.^[^ ^5^ ^]^ This is due to the rapid increase in plastic production since the second half of the 20th century, now exceeding 400,000,000 t year^−1^, while the global recycling rate remains only 9%, causing problems such as marine plastic litter and microplastic pollution.^[^ ^6^ ^]^ Recognizing plastics as a priority sector in its Circular Economy Action Plan, the EU published its European Strategy for Plastics in 2018.^[^ ^7^ ^]^

In 2019, the EU enacted the “Directive on the Reduction of the Impact of Certain Plastic Products on the Environment” (known as the Single‐Use Plastic Directive, or “SUP Directive”).^[^ ^8^ ^]^ This directive introduced mandatory recycled content as a regulatory tool to promote plastic recycling, alongside the prohibition and restriction of single‐use plastics. The directive also sets numerical targets for mandatory recycled content, including 25% for poly(ethylene terephthalate) (PET) bottles by 2025% and 30% for all plastic beverage bottles by 2030. The proposal for a revision of EU legislation on Packaging and Packaging Waste^[^ ^9^ ^]^ (“Packaging Proposal”) suggests that mandatory recycled content should be extended to all plastic packaging, in addition to beverage bottles.

In 2023, the EU released a “Proposal for a Regulation on Circularity Requirements for Vehicle Design and on Management of End‐Of‐Life Vehicles” (“Vehicle Proposal”).^[^ ^10^ ^]^ This proposal mandates that each vehicle type should contain at least 25% plastic recycled from post‐consumer plastic waste, with 25% of that material (6.25% of the total) sourced from recycled end‐of‐life vehicle closed loops.

Plastic recycling (material recovery) technologies are defined in ISO 15270 (2008)^[^ ^11^ ^]^ as mechanical, chemical or feedstock, or biological or organic recycling. However, energy recovery is not included. Mechanical recycling refers to the processing of plastic waste into secondary raw materials or products without significantly changing the chemical structure of the material. Chemical recycling involves the conversion of plastics into monomers or the production of new raw materials by changing the chemical structure of plastic waste through cracking, gasification, or depolymerization, excluding energy recovery and incineration.

There is no description of recycling technologies in the SUP Directive or Packaging Proposal to substantiate mandatory recycled content. In practice, Plastics Europe^[^ ^12^ ^]^ indicated that most recycling technologies for plastic packaging are mechanical recycling, with very few using chemical recycling. In Japan, where chemical recycling is more advanced, 280,000 t of plastic will be recycled by chemical recycling in 2022,^[^ ^13^ ^]^ compared with 1,800,000 t of mechanical recycling. A major part of this will be processed as chemical raw material by a coke oven under the Containers and Packaging Recycling Law.^[^ ^14^ ^]^

The EU Vehicle Proposal highlights concerns from the automotive industry regarding the shortage of recycled plastic materials and emphasizes the need for chemical recycling to meet mandatory recycled content requirements, as noted in an accompanying statement. In addition, an annex document^[^ ^15^ ^]^ to the Vehicle Proposal discusses the possibility of chemical recycling as a necessary response to fulfill these mandates.

In introducing mandatory recycled content, determining a calculation method for the recycled material content and applicable recycling technology is important. A 2022 EU study document^[^ ^16^ ^]^ on calculation rules for recycled material content under the SUP Directive discussed these methods for various recycling technologies. The key points are as follows:

For mechanical recycling, recycled plastic material serves as a secondary raw material in solid form, such as pellets. The recycled content in products can be controlled by adjusting the mixing ratio with virgin material on a lot‐by‐lot or period‐by‐period basis. This implies that labeling products with recycled material content, as mandated by the SUP Directive, Packaging Proposal, and Vehicle Proposal, is relatively easy.

On the other hand, in chemical recycling, other than plants exclusively handling recycled materials, many processes integrate pyrolysis oil derived from recycled plastics into existing facilities, such as steam crackers, which primarily process virgin raw materials from crude oil.^[^ ^17^ ^]^ The key advantage of chemical recycling lies in the production of secondary raw materials of quality comparable to that of virgin raw materials, alongside cost benefits from using existing chemical plants. However, in this case, the amount of recycled material typically constitutes a small portion relative to the amount of virgin raw material derived from crude oil, making it challenging to meet targets such as a 25% mandatory recycled content.

Therefore, a mass balance approach was devised to virtually allocate the content of chemically recycled materials to products. The EU study document examined calculation methods, including the mass balance approach, and its application to construction materials, electronics, and automobiles. While the mass balance approach has been used in other product areas to comply with the mandatory recycled content, challenges such as ensuring value accuracy through third‐party certification persist.^[^ ^18^, ^19^ ^]^ The EU is currently discussing detailed rules for applying the mass balance approach. An EU report on the environmental and economic assessment of plastic recycling^[^ ^20^ ^]^ indicated the advantages of recycling plastics over energy recovery, supporting the view that chemical recycling is preferable to incineration.^[^ ^21^ ^]^ In the future, chemical recycling is expected to become increasingly important for achieving mandatory recycled plastic content targets and accelerating the transition to a circular economy.^[^ ^22^, ^23^ ^]^

Review articles on plastic pyrolysis and product utilization published since 2010 are summarized in **Table** **1**. Research on plastic pyrolysis has mainly focused on polyolefins, such as high‐density polyethylene (HDPE), low‐density polyethylene (LDPE), polypropylene (PP), and polystyrene (PS). Key aspects investigated include the effects of catalysts, reactor types, and process parameters, such as the temperature, residence time, pressure, catalyst‐to‐feed ratio, and type and flow rate of fluidizing gas. In recent years, halogen‐containing plastics, such as polyvinyl chloride (PVC), waste electrical and electronic equipment (WEEE) plastics, and plastics containing brominated flame retardants (BFRs), have received considerable attention as hard‐to‐recycle plastic waste. Achieving carbon neutrality requires recycling these challenging materials. As discussed in the above‐mentioned reviews, pyrolysis is a promising technology capable of converting a broader range of plastic types and quantities into chemical feedstocks compared with mechanical recycling, enabling the reuse of waste plastics as carbon resources.

**Table 1:** Summary of selected review articles on plastic pyrolysis published since 2010.

Year	Paper title	Key points	References
2010	Thermolysis of waste plastics to liquid fuel: A suitable method for plastic waste management and manufacture of value added products—A world prospective	Summarized research on converting plastic waste into liquid fuel via pyrolysis, both with and without catalysts. Discussed future challenges such as scaling up processes, minimizing waste disposal and production costs, and optimizing the recovery of fuel‐range products from mixed plastic waste.	Panda et al.^[^ ^146^ ^]^
2011	A review on tertiary recycling of high‐density polyethylene to fuel	Studied chemical recycling technologies for converting HDPE into fuels. Discussed the pyrolysis mechanisms and kinetics of HDPE. Suggested future challenges, including compiling experimental results and theoretical models of catalytic degradation of common plastics and designing an appropriate process to convert waste plastics into liquid fuels.	Kumar et al.^[^ ^147^ ^]^
2012	Developing advanced catalysts for the conversion of polyolefinic waste plastics into fuels and chemicals	Investigated the bulky nature of polyolefins, which creates steric and diffusive barriers to facilitate zeolite pore penetration, leading to studies on mesoporous materials (Al‐MCMa)‐41 and Al‐SBAb)‐15) and hierarchical zeolites. Discussed catalyst performance, including the acidity and pore structure, in forming aromatic hydrocarbons, particularly BTXc), concluding that zeolites are excellent catalysts for polyolefin cracking. Suggested extra‐large‐pore zeolites, delaminated zeolites, pillared zeolite nanosheets, and hierarchical zeolites as promising candidates for catalytic pyrolysis.	Serrano et al.^[^ ^148^ ^]^
2013	Pyrolysis and dehalogenation of plastics from waste electrical and electronic equipment (WEEE): A review	Investigated the impacts of process configuration, material characteristics, and dehalogenation methods on the pyrolysis of WEEE plastics. Discussed dehalogenation and pyrolysis strategies, including low‐temperature thermally dehalogenation and in‐ and ex situ pyrolysis with catalysts or adsorbents. Highlighted that in situ catalytic pyrolysis prevents oil quality deterioration but increases the overall cost of the catalyst recycling process due to the separation of the pyrolysis and dehalogenation steps.	Yang et al.^[^ ^149^ ^]^
2014	Pyrolysis technologies for municipal solid waste: A review	Pyrolysis plants with adequate capacity to produce energy products are suitable for processing MSWd), provided the quality of the products (char, oil/wax, and combustible gases) is carefully controlled. Summarized key pyrolysis equipment and environmental protection technologies used in pilot, demonstration, and industrial‐scale plants.	Chen et al.^[^ ^150^ ^]^
2015	Current state and future prospects of plastic waste as source of fuel: A review	Explored various pyrolysis reactor types, including batch, fixed‐bed, fluidized‐bed, conical spouted‐bed, and microwave reactors, and the use of supercritical water. Reported on the characteristics of liquid fuels produced from the pyrolysis of single types of plastics, mixed plastics, and municipal plastic waste.	Wong et al.^[^ ^151^ ^]^
2016	A review on pyrolysis of plastic wastes	Discussed the effects of pyrolysis parameters, including reactor type, temperature, residence time, pressure, and catalyst type, on the oil yield and quality. Concluded that pyrolysis products serve as valuable energy fuels, offering a means to reduce the dependence on fossil fuels.	Sharuddin et al.^[^ ^152^ ^]^
2016	Catalytic pyrolysis of plastic waste: A review	Explored the potential of the oil obtained through pyrolysis and catalytic pyrolysis by investigating its physical properties, including its density, viscosity, flash point, HHVe), and boiling range. Concluded that optimizing the process parameters and selecting catalysts based on life cycle assessment and process costs is essential for developing sustainable catalytic pyrolysis processes.	Miandad et al.^[^ ^153^ ^]^
2017	A review on thermal and catalytic pyrolysis of plastic solid waste (PSW)	Discussed the effects of process parameters, including temperature, pressure, and residence time, on product yield and selectivity. Compared the performance of PSWf) treatment methods, such as hydrocracking, gasification, and catalytic pyrolysis. Concluded that PVC pretreatment and further upgrading of the oil quality are necessary to meet the acceptance criteria for oil refinery processes.	Al‐Salem et al.^[^ ^154^ ^]^
2018	An overview of chemical additives present in plastics: migration, release, fate and environmental impact during their use, disposal and recycling	Examined the environmental impacts of plastic additives throughout the use, disposal, and recycling stages of plastic products. Highlighted that various recycling techniques may release PoTSg), such as toxic metals, BFRs, POPs,h), and PAHsi), especially in less developed countries where sorting–reprocessing–recycling conditions are often uncontrolled. Suggested that improved regulatory frameworks and specifications for additives in plastics manufacturing and improved recycling methods for reprocessing plastic waste could reduce environmental and human health impacts in both developed and developing countries.	Hahladakis et al.^[^ ^155^ ^]^
2019	Utilization of waste plastic oil in diesel engines: a review	Investigated the performance of WPOj) for use in diesel engines. Concluded that WPO has significant potential as an alternative to diesel.	Damodharan et al.^[^ ^156^ ^]^
2020	The use of heterogeneous catalysis in the chemical valorization of plastic waste	Discussed the impacts of various heterogeneous catalysts, including SAk), mesoporous silica, zeolite, metal oxides, activated carbon, and clay, on plastic waste pyrolysis. Concluded that significant efforts are needed to chemically convert plastic waste into non‐fuel, reusable, low‐GHGl) emitting products to achieve a circular economy.	Mark et al.^[^ ^157^ ^]^
2021	Thermochemical conversion of plastic waste to fuels: a review	Outlined waste‐to‐energy conversion technologies, such as pyrolysis, liquefaction, and gasification, to convert plastics into clean fuels and chemicals. Discussed the physicochemical properties of fuel products derived from plastic waste, along with the reaction mechanisms, advantages, and technical limitations of each conversion process.	Nanda et al.^[^ ^158^ ^]^
2022	Recent trends in recycling and reusing techniques of different plastic polymers and their composite materials	Discussed the mechanical and chemical recycling, along with the energy recovery, of various plastic‐based composite materials combined with glass fiber and carbon fiber. Reported on recent trends in the production and recycling of composite materials. Highlighted pyrolysis as one of the best methods to recycle composite materials, as it effectively recycles both synthetic fibers and plastics.	Khalid et al.^[^ ^159^ ^]^
2022	Pyrolysis technology for plastic waste recycling: A state‐of‐the‐art review	Discussed recent advances in catalytic pyrolysis techniques for converting plastics into fuels, naphtha, light olefins, and hydrogen, along with their advantages and disadvantages. Conducted techno‐economic and environmental analyses for the pyrolysis of plastic waste. Proposed that pretreatment of PVC in the feedstock and HCl removal from the pyrolysis system as challenges in plastic waste pyrolysis.	Dai et al.^[^ ^160^ ^]^
2023	Catalytic pyrolysis as a platform technology for supporting the circular carbon economy	Identified catalyst deactivation and plant corrosion due to contaminants and heteroatoms as short‐term challenges. Identified the recovery and utilization of by‐products, use of more contaminated mixed waste feedstock, and effective feedstock collection and transportation as long‐term challenges.	Wrasman et al.^[^ ^161^ ^]^
2024	Recent advancements in pyrolysis of halogen‐containing plastics for resource recovery and halogen upcycling: A state‐of‐the‐art review	Reported on achievements and innovations in converting halogen‐containing plastics into valuable products. Highlighted the potential applications of halogen‐doped materials and strategies for upcycling halogens from halogen‐containing wastes.	Ma et al.^[^ ^162^ ^]^

a) Mobil crystalline material;

b) Santa barbara amorphous;

c) Benzene, toluene, and xylene;

d) Municipal solid waste;

e) High heating value;

f) Plastic solid waste;

g) Potentially toxic substances;

h) Persistent organic pollutants;

i) Polycyclic aromatic hydrocarbons;

j) Waste plastic oil;

k) Silica‐alumina;

l) Green house gas.

This review examines the progress of pyrolysis research, with a primary focus on polyolefins such as HDPE, LDPE, PP, and PS. Although numerous studies have been conducted globally since the 1970s on converting these plastics into chemical feedstocks through pyrolysis, there are few examples of industrial‐scale implementation. Accordingly, this review highlights the latest trends in industrial‐scale liquefaction projects. Furthermore, there exists a knowledge gap regarding how laboratory‐level research is—or is not—being translated into industrial‐scale applications. This review addresses the technological disconnect between lab‐scale research and industrial‐scale operations, particularly in the context of pyrolyzing hard‐to‐recycle plastics, such as PVC, BFR‐containing plastics, and PET. Based on these insights, the article proposes future research and development directions to advance plastic pyrolysis technologies and support the transition to a circular economy.

Notably, this review does not focus on copyrolysis with other carbon resources, such as biomass and petroleum, or on pyrolysis studies based on thermal analysis techniques (e.g., thermogravimetric analysis, thermogravimetric analysis‐based kinetic and thermodynamic studies, and pyrolysis‐gas chromatography).

## 2 Latest Trends in Laboratory‐Scale Pyrolytic Liquefaction

**Figure 1:**
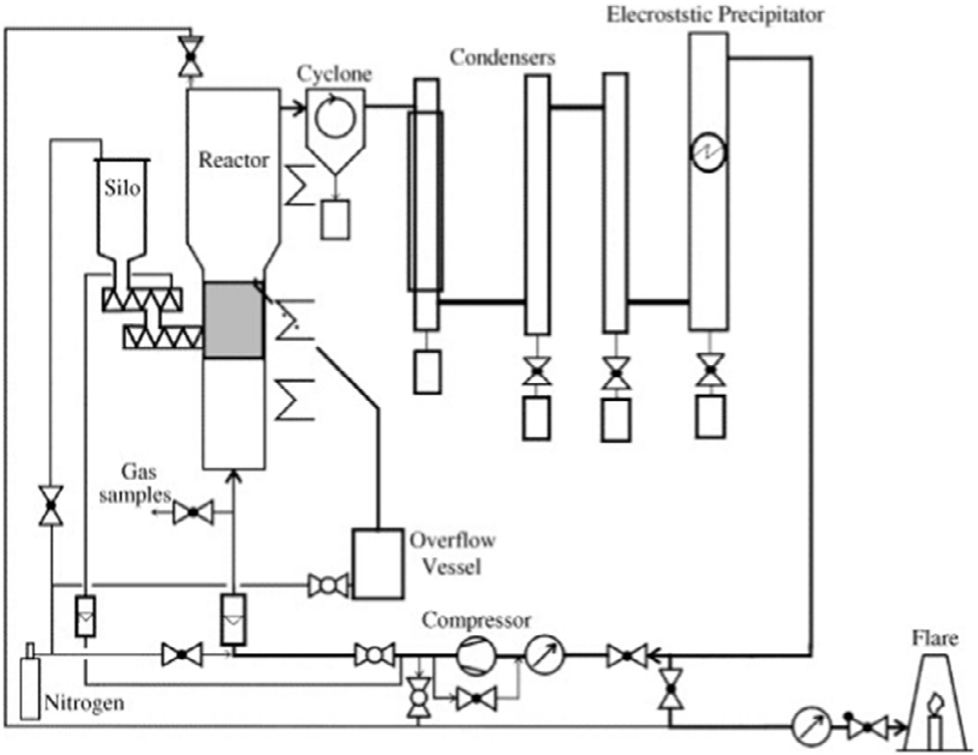
Flow diagram of the fluidized‐bed pyrolysis plant (Hamburg process). [Reproduced with permission.^[^ ^24^ ^]^ Copyright 2004, Elsevier (License number: 5955200217509)].

Pyrolysis is a promising method that simultaneously cleaves multiple chemical bonds in polymers using heat alone, producing chemical feedstocks and fuels such as gas, oil, and solids. The reaction selectivity and product distribution depend on the plastic composition and pyrolysis conditions. Kaminsky et al.^[^ ^24^ ^]^ investigated pyrolysis product distribution using a fluidized‐bed reactor, known as the Hamburg process (**Figure** **1**). Pyrolysis of polyethylene (PE) at 530 °C produced 50.3 wt% oil, mainly consisting of wax, while gas production increased significantly to 55.8 wt% at 760 °C. PS produced 64.9 wt% styrene monomers at 580 °C. In addition, they investigated the pyrolysis of mixed plastics consisting of PE, PP, PS, and traces of PVC at 730 °C,^[^ ^25^ ^]^ which yielded 35.0 wt% gases, 48.4 wt% oil, 14.3 wt% distilled residues, and 2.2 wt% char. The oil produced from pyrolysis contained valuable aromatic compounds, such as benzene, toluene, xylene (BTX), and styrene, contributing to a total yield of 31.4 wt%. In addition, the gases produced had a high calorific value of 50 MJ kg^−1^. When PE‐ and PP‐rich mixed plastics were pyrolyzed in a steam atmosphere, olefins such as 21–29 wt% ethylene, 16–21 wt% propylene, and 5.6–6.6 wt% butadiene were obtained.^[^ ^26^ ^]^ This product distribution was similar to that from the steam cracking of naphtha. Thus, pyrolysis is widely regarded as a viable method for recycling waste plastics into petrochemical feedstocks.

Although pyrolysis is a promising technique for decomposing a wide variety of plastics into gas and oil, the resulting oil and gas are complex mixtures of pyrolysis products. Therefore, many studies have explored the use of catalysts to lower reaction temperatures and improve the yield and selectivity of target fractions, such as light hydrocarbons in the gasoline‐ and naphtha‐range, as well as aromatic hydrocarbons including BTX. This review focuses on selected studies that investigate oil recovery for use as chemical feedstock or fuel, examining the effects of catalysts and pyrolysis conditions on recovery outcomes, as summarized in **Table** **2**.

**Table 2:** Summary of laboratory‐scale catalytic pyrolysis for polyolefin liquefaction in selected studies.

Entry	Year	Plastic (values: wt%)	Reactor	Catalyst	Catalyst characteristics (reported main characteristics)	Sample amount	Temperatures (pyrolysis and catalysis)	Key results	References
1	1997	HDPE, LDPE, linear LDPE, and cross‐linked PE	Semi‐batch (plastic pyrolyzed with catalyst)	SAh) (SiO_2_/Al_2_O_3_ = 5, mol.i) ratio)	BETv) surface area: 420 m^2^ g^−1^	Plastics: 10 g Catalyst: 1.0 g	Pyrolysis and catalysis: 3 °C min^−1^ → 430 °C	The SA catalyst improved oil yields (77–83 wt%) regardless of the type of PE and converted oils, which had a wide carbon number distribution, into the C_5–12_ gasoline range.	Uddin et al.^[^ ^27^ ^]^
2	1997	HDPE	Semi‐batch (plastic pyrolyzed with catalyst)	SA (SiO_2_/Al_2_O_3_ = 5, 0.3, mol. ratio), ZSM‐5 (SiO_2_/Al_2_O_3_ = 75, mol. ratio), and KFSj)‐16	BET surface area; KFS‐16 = 900 m^2^ g^−1^, SA(SiO_2_/Al_2_O_3_ = 5) = 420 m^2^ g^−1^, SA(SiO_2_/Al_2_O_3_ = 0.3) = 270 m^2^ g^−1^, ZSM‐5 = 360 m^2^ g^−1^.	HDPE: 10 g Catalyst: 1.0 g	Pyrolysis and catalysis: 3 °C min^−1 ^→ 430 °C	The catalytic pyrolysis test was repeated four times using KFS‐16, and the oil yield was consistently maintained at ≈80 wt%.	Sakata et al.^[^ ^28^ ^]^
3	1999	HDPE, PP	Semi‐batch (plastic pyrolyzed with catalyst)	SA (SiO_2_/Al_2_O_3_ = 5, 0.3, mol. ratio), ZSM‐5 (SiO_2_/Al_2_O_3_ = 75, mol. ratio), KFS‐16, silicalite, silica gel, and mesoporous silica (FSMk))	BET surface area of FSM: 1030 m^2^ g^−1^	Plastics: 10 g Catalyst: 1.0 g	Pyrolysis and catalysis: 3 °C min^−1^ → 430 °C	The catalysis pyrolysis test using FSM was repeated four times, with oil yields exceeding 80% for HDPE and 83% for PP from the second to the fourth test.	Sakata et al.^[^ ^29^ ^]^
4	1999	PE	Two‐stage FxBRe) (first: plastic melter, second: catalytic bed)	SA (13.5 wt% Al_2_O_3_) and HZSM‐5 (Si/Al = 11, atl). ratio)	–	Space time: 4–18 g‐cat min g‐PE^−1^ Catalyst: 0.2–0.3 g	Pyrolysis and catalysis: 375–425 °C	The oil yield was 58.8 wt%, with aromatics and benzene contents of 25.2 and 0.9 wt%, respectively.	Uemichi et al.^[^ ^44^ ^]^
5	2001	HDPE, LDPE, PP	Two‐stage FxBR (first: plastic melter, second: catalytic bed)	Ga‐loaded HZSM‐5 (Si/Ga = 25, at. ratio)	–	1–25 g‐cat min g‐PP^−1^	Pyrolysis and catalysis: 375–550 °C	In LDPE, 71.6 wt% aromatic hydrocarbons (including 61.8 wt% BTX) were obtained at 525 °C, while in PP, the BTX yield exceeded 60 wt% at 550 °C.	Takuma et al.^[^ ^38^ ^]^
6	2002	PE	Two‐stage FxBR (first: plastic melter, second: catalytic bed)	ZSM‐5 (SiO_2_/Al_2_O_3_ = 50, mol. ratio) and Y‐zeolite (SiO_2_/Al_2_O_3_ = 11, mol. ratio)	ZSM‐5: particle size = 2 mm, average pore size = 0.55 nm, pore volume = 0.48 m^3^ kg^−1^, surface area = 300 m^2^ g^−1^, bulk density = 0.72 kg m^−3^. Y‐zeolite: particle size = 2 mm, pore size = 0.74 nm, pore volume = 0.64 m^3^ kg^−1^, surface area = 440 m^2^ g^−1^, bulk density = 0.61 kg m^−3^.	PE: – Catalyst: 10 g	Pyrolysis: 500 °C Catalysis: 400–600 °C	The toluene content was 12.96 wt% and the ethylbenzene content was 11.38 wt% in the oil at a catalyst bed temperature of 600 °C when Y‐zeolite was used.	Bagri and Williams^[^ ^30^ ^]^
7	2009	HDPE	CSBRf)	CBV3024E,m) CBV600,n) and CP811E‐75o)	HZSM‐5: BET surface area = 182 m^2^ g^−1^, *B*/*L* w) ratio = 2.32. HY: BET surface area = 231 m^2^ g^−1^, *B*/*L* ratio = 1.01. Hβ: BET surface area = 221 m^2^ g^−1^, *B*/*L* ratio = 1.51.	HDPE: 1 g min^−1^ (Total: 900 g) Catalyst: 30 g	Pyrolysis and catalysis: 500 °C	The yields of the individual products were 28 wt% propylene, 20 wt% butene, and 10 wt% ethylene when HZSM‐5 was used.	Elordi et al.^[^ ^31^ ^]^
8	2011	MPa) (40 HDPE, 35 PP, 18 PS, 4 PET, and 3 PVC)	Semi‐batch (plastic pyrolyzed with catalyst)	ZSM‐5 (SiO_2_/Al_2_O_3_ = 50)	BET surface area = 412 m^2^ g^−1^, micropore volume = 0.1 cm^3^ g^−1^, total pore volume = 0.4 cm^3^ g^−1^, micropore area = 346.1 m^2^ g^−1^.	10 g of zeolite was mixed with a 100 g plastic sample	Pyrolysis and catalysis: 440 °C	In fresh ZSM‐5, toluene was 12.3 area%, dimethyl‐heptene 1.8 area%, ethyl‐benzene 10.6 area%, xylenes 10.1 area%, and styrene 31.4 area%.	López et al.^[^ ^34^ ^]^
9	2012	MP (46 LDPE, 30 HDPE, and 24 PP)	FBRg)	Commercial Ziegler–Natta (TiCl/MgCl_2_, TiCl_4_ = 5–16 wt%)	Settled apparent density of 400–700 kg m^−3^.	Plastic total input: 0.964–1.993 kg (0.24–0.91 kg h^−1^) Catalyst supplied: 1%	Pyrolysis and catalysis: 500, 650 °C	At 650 °C, the gas product yield was 54.3 wt%, with higher yields of ethylene (22.3 wt%), propylene (21.1 wt%), and methane (21.0 wt%) compared to non‐catalytic pyrolysis.	Donaj et al.^[^ ^54^ ^]^
10	2015	LDPE	Microwave pyrolysis + FxBR (catalytic bed)	ZSM‐5 (SiO_2_/Al_2_O_3_ = 50)	BET surface area = 412 m^2^ g^−1^, micropore volume = 0.1 cm^3^ g^−1^, total pore volume = 0.4 cm^3^ g^−1^, micropore area = 346.1 m^2^ g^−1^, total acidity = 0.18 mmol NH_3_ g^−1^.	LDPE: 20 g Catalyst: 4.27–15.15 g	Pyrolysis: 400 °C Catalysis: 250–450 °C	The monocyclic aromatic hydrocarbons in the pyrolysis oil ranged from 74.73 to 88.49 area%.	Zhang et al.^[^ ^35^ ^]^
11	2017	HDPE	FxBR	Sulfated zirconia	BET surface area = 116 m^2^ g^−1^, micropore volume = 0.0011 cm^3^ g^−1^, pore volume = 0.21 cm^3^ g^−1^, micropore area = 4.22 m^2^ g^−1^, SO_3_ = 6.64 mol%, total acidity = 337.6 μmol NH_3_ g^−1^.	HDPE: 2 g Catalyst: 0.2 g	Pyrolysis and catalysis: 370–430 °C	At 380 °C, the oil yield was 38.5 wt%, and the oil composition consisted of 58% aromatic compounds, 21% olefins, 16% paraffins, and 5% naphthalene.	Almustapha et al.^[^ ^48^ ^]^
12	2017	HDPE	Two‐stage FxBR (first: plastic melter, second: catalytic bed)	ZSM‐5 (Si/Al = 20, at. ratio) and MCM‐41 (Si/Al = 4, at. ratio)	ZSM‐5: BET surface area = 266 m^2^ g^−1^, micropore volume = 0.23 cm^3^ g^−1^, mesopore volume = 0.12 cm^3^ g^−1^, average pore size = 5.2 nm. MCM‐41: BET surface area = 799 m^2^ g^−1^, micropore volume = 0.38 cm^3^ g^−1^, mesopore volume = 0.33 cm^3^ g^−1^, average pore width = 3.95 nm.	HDPE: 2 g Catalyst: 4 g	Pyrolysis and catalysis: 500 °C	The catalyst weight ratio of ZSM‐5 to MCM‐41 (1:1) produced an oil yield of more than 80 wt%, with aromatic and gasoline‐range hydrocarbons (C_8–12_) accounting for 95.85 and 97.72 area%, respectively.	Ratnasari et al.^[^ ^45^ ^]^
13	2017	MP (42 HDPE, 35 PP, 18 PS, and 5 PET)	Semi‐batch (plastic pyrolyzed with catalyst)	Al‐, Fe‐, Ti‐, and Zr‐pillared clays	Fe‐PILC: BET surface area = 215 m^2^ g^−1^, micropore volume = 0.052 cm^3^ g^−1^, total pore volume = 0.290 cm^3^ g^−1^, surface acidity = 179.3 μmol pyridine g‐catalyst^−1^ (573 K), 43.8 μmol 2,6 DMPYx) g‐catalyst^−1^ (573 K).	The plastic sample weighed ≈10 g, and the mass ratio of catalyst to plastics was 1:10	Pyrolysis and catalysis: 300 °C → 10 °C min → 500 °C	Fe‐PILC had an oil yield of 79.3 wt%, of which 80.5% was diesel fraction (C_13–19_). The oil components consisted of 11.0 area% light aromatic hydrocarbons (≤C_13_) and 33.5 area% heavy aromatic hydrocarbons (>C_13_).	Li et al.^[^ ^55^ ^]^
14	2017	LDPE	Microwave pyrolysis + FxBR (catalytic bed)	MgO	–	HDPE: 15 g MgO to LDPE ratios: 1/3_–_1/15	Pyrolysis: 350–500 °C Catalysis: 350–550 °C	The oil yields ranged from 24.2 to 38.5 wt%, while the hydrocarbon content of the gasoline fraction was up to 96.0% (39.7% monocyclic aromatics, 56.3% C_5–12_ aliphatics).	Fan et al.^[^ ^50^ ^]^
15	2019	LDPE	Microwave pyrolysis + FxBR (catalytic bed)	NiO and HY zeolite (Si/Al = 15, at. ratio)	Specific surface area of HY = 750 m^2^ g^−1^	LDPE: 15 g HY‐to‐LDPE Mass ratios: 1/5–1/20 NiO‐to‐HY Mass ratio: 1/3–1/15	Pyrolysis: 450–600 °C Catalysis: 350–500 °C	At a pyrolysis temperature of 500 °C, a catalyst temperature of 450 °C, and an HY/LDPE ratio of 1:10, the oil yield was 56.54 wt%. The oil components were 46.61% aromatics, 10.97% n‐alkanes, and 2.94% n‐alkenes.	Ding et al.^[^ ^56^ ^]^
16	2020	HDPE	FxBR	Sulfated zirconia modified with calcium carbide	BET surface area = 23.1 m^2^ g^−1^, pore volume = 0.04 cm^3^ g^−1^, micropore area =<0.1 m^2^ g^−1^, SO_3_ = 6.64 mol%, total acidity = 23.4 μmol NH_3_ g^−1^.	HDPE: 5 g Catalyst: 5 g	Pyrolysis and catalysis: 410 °C	The oil yield was 66 wt%, and the oil composition consisted of 74% olefins, 23% naphthenes, 3% paraffins, and less than 0.1% aromatic.	Almustapha et al.^[^ ^66^ ^]^
17	2020	PE	FxBR	SiO_2_, SA, Si‐MFI, and Si/Al‐MFI	ZSM‐5: BET surface area = 387 m^2^ g^−1^, micropore volume = 0.15 cm^3^ g^−1^, total pore volume = 0.17 cm^3^ g^−1^, total acidity = 0.638 mmol NH_3_ g^−1^.	Catalyst weight: 10 wt%	Pyrolysis and catalysis: 450 °C	The oil yield was 36 wt% when HZSM‐5 was used, with light fractions of C_3–5_ accounting for 55% of the oil. The percentage of aromatic compounds in the oil was 25%.	Klaimy et al.^[^ ^32^ ^]^
18	2021	HDPE, LDPE, PP, HIPSb), GPPSc)	Two‐stage FxBR (first: plastic melter, second: catalytic bed)	Fe/Al_2_O_3_	BET surface area = 96.78 m^2^ g^−1^, total pore volume = 0.62 cm^3^ g^−1^, average diameters catalyst metal particles = 25.78 nm	Plastics: 1 g Catalyst: 0.5 g	Pyrolysis: 500 °C Catalysis: 800 °C	The oil yield was about 20 wt% with a carbon distribution of C_6–22_. In terms of the components in the oil, PP had a significantly higher proportion of naphthalene (C_10_) at 27.2%, followed by pyrene (C_16_) at 15.7%, anthracene (C_14_) at 15.3%, and biphenyl (C_12_) at 12.7%.	Cai et al.^[^ ^51^ ^]^
19	2021	wd) PP	Two‐stage FxBR (first: plastic melter, second: catalytic bed)	Fe/Al_2_O_3_ (1/20, 1/10, 1/5, 1/2, 1/1, 2/1)	Fe/Al_2_O_3_ (1/2): BET surface area = 96.78 m^2^ g^−1^, total pore volume = 0.62 cm^3^ g^−1^, average diameters catalyst metal particles = 25.78 nm	PP: 1 g Catalyst: 0.5 g	Pyrolysis: 500 °C Catalysis: 800 °C	For Fe/Al_2_O_3_(1/2), the naphthalene content in the oil was the highest, reaching 71.38 area%.	Cai et al.^[^ ^52^ ^]^
20	2021	HDPE	CSBR	Spent FCCp)	BET surface area = 143 m^2^ g^−1^, total acidity = 124 μmol NH_3_ g^−1^.	HDPE: 1 g/minCatalyst: 7–45 g	Pyrolysis and catalysis: 475–600 °C	The maximum yield of C_5–11_ hydrocarbons (61.2 wt%) was obtained with 15 g of catalyst at a catalytic pyrolysis temperature of 500 °C.	Orozco et al.^[^ ^46^ ^]^
21	2021	PP	Semi‐batch (plastic pyrolyzed with catalyst)	Fresh FCC,, FCC‐NZq), Spent FCC(ECATr))	Fresh FCC: BET surface area = 261.1 m^2^ g^−1^, micropore volume = 84 μL g^−1^, mesopore volume = 70.3 μL g^−1^. FCC‐NZ: BET surface area = 83.6 m^2^ g^−1^, micropore volume = 3.9 μL g^−1^, mesopore volume = 82.4 μL g^−1^. Spent FCC (ECAT): BET surface area = 183.2 m^2^ g^−1^, micropore volume = 49.4 μL g^−1^, mesopore volume = 88.7 μL g^−1^.	PP: 2.5 g Catalyst: 1.25 g	Pyrolysis and catalysis: 450 °C	The yield of aromatic compounds was about 20% and increased in the following order: spent FCC < FCC < FCC‐NZ.	Vollmer et al.^[^ ^47^ ^]^
22	2021	LDPE	FxBR	Kaolin (325, 800 and 1250 mesh)	Kaolin (1250 mesh): BET surface area = 3.929 m^2^ g^−1^, average pore diameter = 3.812 nm, total pore volume = 0.014 cm^3^ g^−1^	LDPE: 90 g Catalyst: 10 g	Pyrolysis and catalysis: 600 °C	Regardless of the mesh size of Kalion, the oil yield remained around 70 wt%. In the case of Kalion (1250 mesh), the content of aromatic hydrocarbons–particularly polycyclic aromatic hydrocarbons– in the oil increased with repeated use.	Luo et al.^[^ ^57^ ^]^
23	2022	LDPE	FxBR, Two‐stage FxBR (first: plastic melter, second: catalytic bed)	HZSM‐5 (Si/Al = 12, 19, 108, at. ratio)	Si/Al = 12: BET surface area = 411 m^2^ g^−1^, micropore volume = 0.127 cm^3^ g^−1^, total acidity = 1.407 mmol NH_3_/g. Si/Al = 19: BET surface area = 412 m^2^ g^−1^, micropore volume = 0.136 cm^3^ g^−1^, total acidity = 0.959 mmol NH_3_ g^−1^. Si/Al = 108: BET surface area = 368 m^2^ g^−1^, micropore volume = 0.140 cm^3^ g^−1^, total acidity = 0.161 mmol NH_3_ g^−1^.	LDPE: 10 g (one‐stage), 4 g (two‐stage) Catalyst‐to‐LDPE ratios: 0.5/10–1/10	Pyrolysis and catalysis: 500 °C	For Si/Al = 12, the aromatic hydrocarbons in the oil were about 55 area% in in‐situ mode and about 77 area% in ex situ mode.	Inayat et al.^[^ ^33^ ^]^
24	2022	wHDPE	Microwave pyrolysis + FxBR (catalytic bed)	ZSM‐5 (Si/Al = 80, 280, 1500) powders and a ZSM‐5 (Si/Al = ^−1^80) pellet containing 30 wt% pseudo‐boehmite binders: ZSM‐5 (Si/Al = 80, 280, and 1500)‐coated silicon carbide (ZSM‐5 loading: 3.4–15 wt%) *Si/AI: molar ratio	ZSM‐5 (Si/Al = 80) coated SiC; BET surface area = 462.7 m^2^ g^−1^, micropore area = 291.9 m^2^ g^−1^, micropore volume = 0.12 cm^3^ g^−1^, mesopore volume = 0.16 cm^3^ g^−1^, macropore volume = 0.07 cm^3^ g^−1^, acidity = 0.53 mmol NH_3_ g^−1^.	HDPE: 120 g h^−1^ (total run time : 6 h)	Pyrolysis: 500 °C Catalysis: 350–450 °C	The gasoline‐range (C_5–12_) hydrocarbons were 28.3%, and monocyclic aromatics were 24.4% in the oil when ZSM‐5 coated SiC with Si/Al = 80 was used.	Zhou et al.^[^ ^42^ ^]^
25	2022	HDPE	Microwave pyrolysis + FxBR (catalytic bed)	ZSM‐5 (SiO_2_/Al_2_O_3_ = 25, 80, 280, 800, 1500 mol. ratio)	ZSM‐5 (SiO_2_/Al_2_O_3_ = 800): BET surface area = 299.3 m^2 ^g^−1^, micropore volume = 0.111 cm^3 ^g^−1^, total pore volume = 0.197 cm^3 ^g^−1^, total acidity = 0.254 mmol NH_3_ g^−1^.	HDPE: 42 g h^−1^ Catalyst: 6 g	Pyrolysis: 500 °C Catalysis: 340–500 °C	The oil yield was about 50 wt% regardless of the silica‐alumina ratio. However, at silica‐alumina ratios of 800 and 1500, olefins (C_5–12_) were the main products, and aromatic selectivity was greatly reduced.	Dai et al.^[^ ^36^ ^]^
26	2022	LDPE	Autoclave	Al_2_O_3_, Pt/Al_2_O_3_, SiO_2_, Pt/SiO_2_, Pt‐0.25Fe/Al_2_O_3_, Pt‐Fe/Al_2_O_3_	Pt‐0.25Fe/Al_2_O_3_: BET surface area = 140 m^2^ g^−1^, total pore volume = 0.262 cm^3^ g^−1^,	LDPE: 1 g Catalyst: 1 g	Pyrolysis and catalysis: 330 °C	Pt‐0.25Fe/Al_2_O_3_ showed the highest oil yield of 59.3 wt%. The selectivity of the products in the oil was 29.0% for gasoline fraction (C_7–12_), 52.1% for jet fuel fraction (C_9–16_), and 55.2% for diesel fraction (C_9–22_). Oil yield and product selectivity remained unchanged after catalyst regeneration.	Chen et al.^[^ ^53^ ^]^
27	2023	LDPE	Two‐stage FxBR (first: pyrolysis, second: catalytic bed)	HZSM‐5, Ga/HZSM‐5, and Ga/P/HZSM‐5 (3 wt%‐Ga and 3 wt%‐P, SiO_2_/Al_2_O_3_ = 25, mol. ratio)	Ga/P/HZSM‐5: BET surface area = 300.5 m^2^ g^−1^, micropore volume = 0.0937 cm^3^ g^−1^, total pore volume = 0.1197 cm^3^ g^−1^, mesopore volume = 0.026 cm^3^ g^−1^.	LDPE: 10 g Catalyst: 4 g	Pyrolysis: 500 °C Catalysis: 450–600 °C	Ga/P/HZSM‐5 showed high selectivity for monoaromatic hydrocarbons (MAH) at 90.7% and for benzene, toluene, ethylbenzene, and xylene (BTEX) at 77.6%.	Zhang et al.^[^ ^39^ ^]^
28	2023	HDPE	Two‐stage FxBR (first: pyrolysis, second: catalytic bed)	Zn/HZSM‐5‐(IWIs)) and Zn/HZSM‐5‐(IEt)) (SiO_2_/Al_2_O_3_ = 25, 50, 100, mol. ratio)	2 wt%‐Zn/HZSM‐5‐(IWI, SiO_2_/Al_2_O_3_ = 25): BET surface area = 363 m^2^ g^−1^, micropore volume = 0.18 mL g^−1^, mesopore volume = 0.13 mL g^−1^, total acidity = 1.62 mmol NH_3_ g^−1^	HDPE: 1.0 g Catalyst: 0.35 g	Pyrolysis and catalysis: 500 °C	The aromatic yield and BTX selectivity were 48% and 93%, respectively, when 2 wt%‐Zn/HZSM‐5(IWI)(SiO_2_/Al_2_O_3_ = 25) was used.	Qian et al.^[^ ^40^ ^]^
29	2024	HDPE	FxBR	USYu), Fe_2_O_3_/USY, Fe_3_O_4_ + Fe(dominantly)/USY and Fe/USY	Fe/USY: BET surface area = 500 m^2^ g^−1^, micropore volume = 0.200 mL g^−1^, acidity = 855 μmol NH_3_ g^−1^	HDPE: 10 g Catalyst: 1 g	Pyrolysis and catalysis: 500 °C	The oil composition obtained using Fe/USY was 53.9% alkanes, 17.8% alkenes, and 28.3% aromatics.	Chen et al.^[^ ^41^ ^]^
30	2024	wPP, wLDPE, wHDPE, and wPS	FxBR	h‐ZSM‐5 zeolite	BET surface area = 295 m^2^ g^−1^, micropore volume = 0.15 cm^3^ g^−1^, total pore volume = 0.38 cm^3^ g^−1^, mesopore volume = 0.23 cm^3^ g^−1^.	Plastics: 20 g Catalyst: 2 g	Pyrolysis and catalysis: 400–430 °C	Catalytic pyrolysis of PS increased the oil yield from 53.4% (pyrolysis) to 63%, with the highest aromatic content in the oil (75%) compared to other plastics.	Subhashini and Mondal^[^ ^43^ ^]^
31	2025	PP	Two‐stage FBR (first: pyrolysis, second: catalytic bed)	H‐ZSM‐5(SiO_2_/Al_2_O_3_ = 38)	BET surface area = 342 m^2^ g^−1^, pore volume = 0.396 cm^3^ g^−1^, total acidity = 548 μmol NH_3_ g^−1^	PP: 150 g h^−1^ Catalyst: 100 g	Pyrolysis: 500–700 °C Catalysis: 500 °C	The maximum BTX yield was 22.3 wt% at a pyrolysis temperature of 550 °C. Toluene had the highest selectivity of 50 mol% among the BTX regardless of the pyrolysis temperature.	Wang^[^ ^37^ ^]^
32	2025	wMP, wHDPE, wLDPE and wPP	Microwave reactor	ZnO	BET surface area = 3.117 m^2^ g^−1^, average particle size = 30.9 μm, average pore diameter = 3.77 nm.	Plastics: 40–50 g Catalyst: 10 g	Pyrolysis and catalysis: 280 °C	In 50 cycles of catalytic reforming at a reaction temperature of 280 °C, reaction time of 30 min, and ZnO: wPP = 10 g: 50 g, the oil yield was maintained ≈80 wt%.	Zhao^[^ ^58^ ^]^

a) Mixed plastic;

b) High impact polystyrene;

c) General purpose polystyrene;

d) Waste;

e) Fixed‐bed reactor;

f) Conical spouted‐bed reactor;

g) Fluidized‐bed reactor;

h) Silica‐alumina;

i) Molar;

j) Kanite‐derived folded silica;

k) Folded‐sheet mesoporous material;

l) Atomic;

m) 25 wt% HZSM‐5 zeolite (SiO_2_/Al_2_O_3_ = 30 mol. ratio), 30 wt% bentonite, 45 wt% inert alumina;

n) 25 wt% HY zeolite (SiO_2_/Al_2_O_3_ = 5.2, mol. ratio), 30 wt% bentonite, 45 wt% inert alumina;

o) 25 wt% of Hβ zeolite (SiO_2_/Al_2_O_3_ = 75, mol. ratio), 30 wt% bentonite, 45 wt% inert alumina;

p) Fluidized catalytic cracking;

q) FCC catalyst without zeolite;

r) Equilibrium catalyst;

s) Incipient wetness impregnation;

t) Aqueous‐phase ion exchange;

u) Ultrastable Y‐zeolite;

v) Brunauer–Emmett–Teller;

w) Brönsted/Lewis site ratio;

x) Dimethylpyridine.

Silica‐alumina (SA) catalysts, commonly known as medium‐acid catalysts, have been used in the pyrolysis of PE and PP. Sakata et al.^[^ ^27^, ^28^, ^29^ ^]^ comprehensively investigated the catalytic effects of SA on the pyrolysis of HDPE, LDPE, linear LDPE, and cross‐linked PE (Table 2, entries 1–3). In the absence of a catalyst, pyrolysis of HDPE and cross‐linked PE at 430 °C produced substantial amounts of wax and 58–63 wt% oil. In contrast, LDPE and linear LDPE produced lower wax contents and higher oil yields (76–77 wt%). These results indicated that branched PEs are more suitable for oil production than their linear‐chain counterparts. The product oils exhibited a broad carbon number distribution (C_5–25_, boiling point range: 36–405 °C). In contrast, the SA catalyst improved oil yields from all PEs and transformed the oils with a broad carbon number distribution into the gasoline range of C_5–12_ (boiling point range: 36–216 °C). Thus, it was concluded that SA, with its moderate acidity, is a promising catalyst capable of lightening product oils without causing significant aromatization. In addition, the stability of the mesoporous silica (KFS‐16 and folded‐sheet mesoporous material (FSM)) was evaluated during the catalytic pyrolysis of PE and PP. Over four repeated catalytic pyrolysis tests, oil yields of ≈80 wt% were consistently maintained for all catalysts, with minimal deactivation observed for FSM. Sakata et al.^[^ ^29^ ^]^ (Table 2, entry 3) concluded that the ability of the FSM to suppress coke precipitation and enhance decomposition was attributed to its large hexagonal pore structure. This structure allowed pyrolysis products to remain within the pores and promoted decomposition.

**Figure 2:**
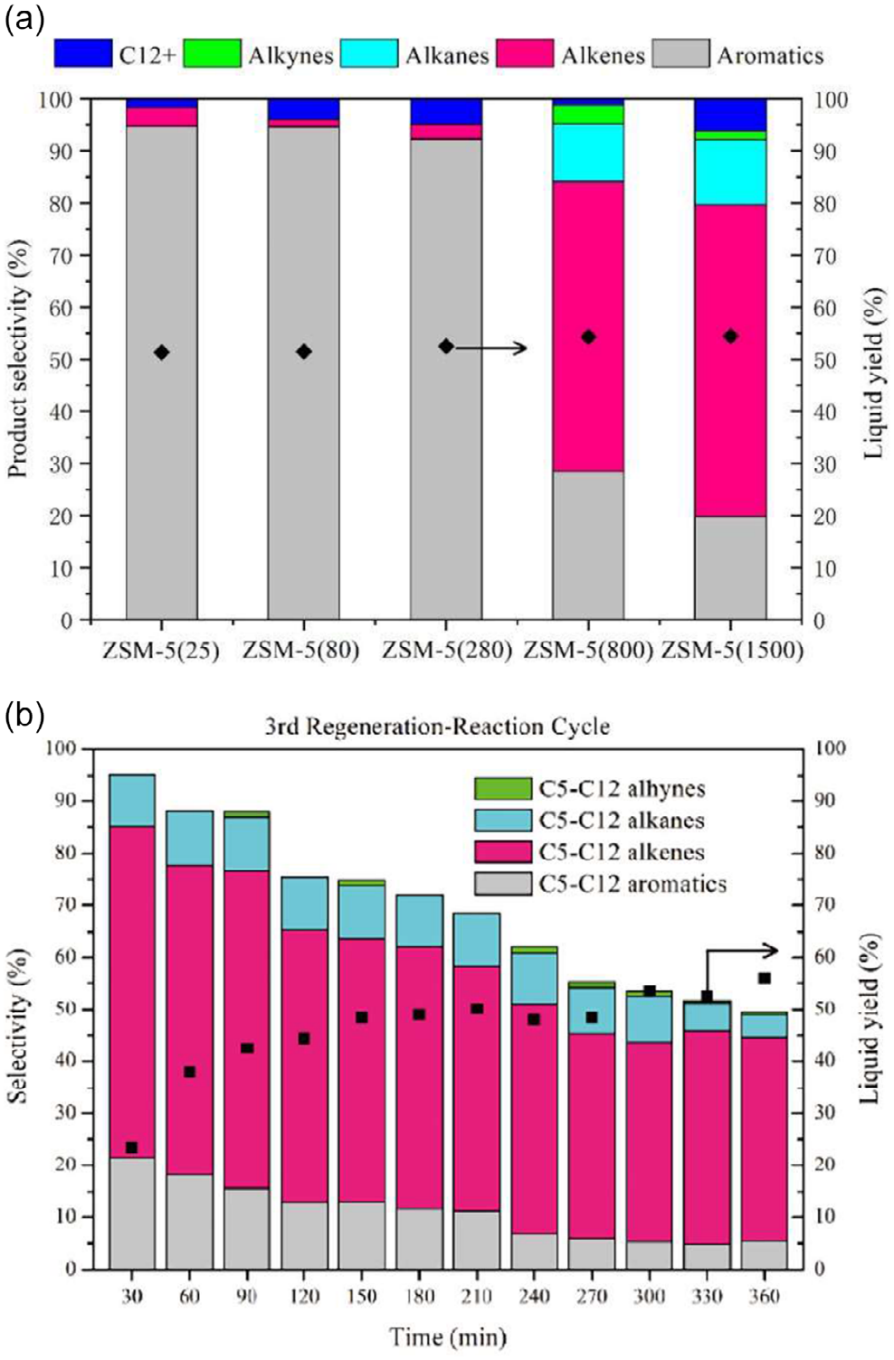
a) Product selectivity and liquid yield over ZSM‐5 zeolites with various SiO_2_/Al_2_O_3_ ratios. b) Product selectivity and liquid yield as a function of time on stream over regenerated ZSM‐5(800) catalysts. Reproduced with permission.^[^ ^36^ ^]^ Copyright 2022, Elsevier [License number: 6015100134635].

Zeolite catalysts, which are widely used in industrial applications, have been extensively studied for their role in the catalytic pyrolysis of plastics. Commonly employed zeolites include ZSM‐5, Y‐type, β‐type, and Ultrastable Y‐zeolite (USY)‐type catalysts. Bagri and Williams^[^ ^30^ ^]^ examined the catalytic pyrolysis of PE in a two‐stage fixed‐bed reactor using ZSM‐5 and Y‐zeolites (Table 2, entry 6). The main aromatic hydrocarbons produced in the oil were toluene, ethylbenzene, and xylene, with their aromatic content increasing as the catalyst temperature rose. They concluded that the higher concentration of aromatic compounds in the oil when using Y‐zeolite was owing to its larger pore size, higher surface acidity, and greater surface area compared with those of ZSM‐5. Elordi et al.^[^ ^31^ ^]^ (Table 2, entry 7) studied the continuous catalytic cracking of HDPE in a conical spouted‐bed reactor using commercial HZSM‐5 (CBV3024E), HY (CBV600), and Hβ (CP811E‐75). The reactor was operated at 500 °C, with 900 g of plastic fed at a rate of 1 g min^−1^. Using HZSM‐5, the gas yield reached 74%, with an impressive light olefin yield of 70%. In contrast, HY zeolite produced a liquid yield of 63%, with the liquid's boiling point falling within the gasoline region. The product distribution obtained with the Hβ zeolite was evenly split between gas (48%) and oil (50%). These results indicated that HZSM‐5 is selective for light olefins, while Hβ and HY zeolite‐based catalysts yield high amounts of nonaromatic products (C_5–11_). However, the main limitation of these catalysts is their deactivation owing to coke deposition. Klaimy et al.^[^ ^32^ ^]^ (Table 2, entry 17) investigated the effect of catalyst acidity on the composition of pyrolysis products using amorphous silica (0.045 mmol‐NH_3_ g^−1^), SA (0.209 mmol‐NH_3_ g^−1^), silicalite (0.017 mmol‐NH_3_ g^−1^), and ZSM‐5 (0.638 mmol‐NH_3_ g^−1^). When PE was pyrolyzed at 450 °C with these catalysts, the amounts of low‐molecular‐weight and aromatic compounds in the pyrolysis products increased in the order of ZSM‐5 > silicalite > SA > amorphous silica. Inatay et al.^[^ ^33^ ^]^ (Table 2, entry 23) investigated the catalytic pyrolysis of LDPE using HZSM‐5 with Si/Al ratios of 12, 19, and 108. The experiments were conducted by mixing LDPE and the catalyst in an in situ mode and in a two‐stage fixed‐bed reactor for the ex situ mode. When the Si/Al ratio was 12 (acid sites: 1.407 mmol g^−1^), the aromatic hydrocarbons in the oil reached ≈55 area% in the in situ mode, increasing to ≈77 area% in the ex situ mode. However, with a Si/Al ratio of 108 (acidic sites: 0.161 mmol g^−1^), the catalytic pyrolysis mode showed no significant impact on the product composition. These results were attributed to the acidity of the catalyst and shorter contact time between pyrolysis vapors and catalyst particles due to the lower acid site density. López et al.^[^ ^34^ ^]^ (Table 2, entry 8) evaluated the deactivation and regeneration of ZSM‐5 (SiO_2_/Al_2_O_3_ = 50) during the catalytic pyrolysis of mixed plastics containing 4 wt% PET and 3 wt% PVC. The catalytic pyrolysis of 100 g of mixed plastic (with 10 g of ZSM‐5) was performed in a semi‐batch reactor at 440 °C. The aromatic content in the oil decreased significantly from 95.2 area% with fresh ZSM‐5 to 78.4 area% when using spent ZSM‐5. Regenerated ZSM‐5 (via combustion at 550 °C) showed 97.4 area% aromatic content in the oil, which was similar to that obtained using the fresh catalyst. In addition, the effects of microwave heating on pyrolysate recovery have been reported. Zhang et al.^[^ ^35^ ^]^ (Table 2, entry 10) investigated the microwave‐assisted catalytic pyrolysis of LDPE using ZSM‐5. At 450 °C and a feedstock‐to‐catalyst ratio of 2, a maximum oil yield of 32.58 wt% was obtained. Increasing the feedstock‐to‐catalyst ratio to 4 resulted in monocyclic aromatic hydrocarbons in the gasoline range reaching 88.49 area% in the oil. Recently, ZSM‐5 catalysts with high silica‐to‐alumina ratios has been investigated. Dai et al.^[^ ^36^ ^]^ (Table 2, entry 25) performed catalytic pyrolysis of HDPE in a two‐stage fixed‐bed reactor using ZSM‐5 with silica‐to‐alumina ratios ranging from 25 to 1500. The oil yield was about 50 wt% regardless of the silica‐to‐alumina ratio. However, at ratios of 800 and 1500, olefins (C_5–12_) were the main products, and aromatic selectivity was significantly reduced (**Figure** **2**a). Additionally, a catalyst regeneration cycle test was conducted using ZSM‐5 with a silica‐to‐alumina ratios of 800. The results showed that the catalyst maintained its product selectivity and oil yield without significant deterioration (Figure 2b). Wang et al.^[^ ^37^ ^]^ (Table 2, entry 31) investigated BTX selectivity during the catalytic pyrolysis of PP in a two‐stage fluidized bed reactor. By arranging the fluidized bed reactors in series, efficient heat and mass transfer were achieved for both pyrolysis and catalytic reforming stages, along with independent temperature control for each reactor. The maximum BTX yield reached 22.3 wt% when the pyrolysis temperature of PP was set at 550 °C. Among the BTX components, toluene exhibited the highest selectivity, accounting for 50 mol% regardless of the pyrolysis temperature. The study also examined the effects of fluidizing gas flow rate and catalyst particle size. Lower nitrogen flow rates and smaller catalyst particles let to higher BTX yields. During continuous operation over a 10 h period, the BTX yield slightly decreased but remained above 20 wt%.

Several research groups have reported the effectiveness of metal‐loaded and structurally modified zeolite catalysts. Uemichi et al.^[^ ^38^ ^]^ studied the two‐step catalytic pyrolysis of HDPE, LDPE, and PP using Ga‐loaded HZSM‐5 (Table 2, entry 5). In the absence of a catalyst, wax was the main product for all plastics; however, catalytic pyrolysis improves the selectivity for aromatic hydrocarbons. For LDPE, 71.6 wt% aromatic hydrocarbons (including 61.8 wt% BTX) were obtained at 525 °C. For PP, the BTX yield increased with higher catalyst pyrolysis temperatures, exceeding 60 wt% at 550 °C. In addition, the BTX yield increased with the catalyst‐to‐feed ratio, reaching over 70 wt% at 25 g‐cat min g‐PP^−1^. The stability of Ga‐loaded HZSM‐5 was also examined through repeated catalytic pyrolysis of LDPE at 525 °C for 15 min. The results showed that the aromatic hydrocarbon yield remained above 60 wt% after a cumulative operating time of 180 min, confirming the high stability of Ga‐loaded HZSM‐5 in the catalytic pyrolysis of LDPE. Zhang et al.^[^ ^39^ ^]^ (Table 2, entry 27) studied the effects of Ga/P/HZSM‐5 on the catalytic pyrolysis of LDPE in a two‐stage fixed‐bed reactor. The maximum oil yield was 42.1%, and G/P/HZSM‐5 exhibited a high selectivity for monoaromatic hydrocarbon (MAH) (90.7%) and benzene, toluene, ethylbenzene, and xylene (BTEX) (77.6%). Compared with HZSM‐5, Ga/HZSM‐5 benefited from aromatic formation owing to its enhanced dehydrogenation capacity. Furthermore, introducing P into the catalyst was beneficial for modulating the acidity of Ga/HZSM‐5, producing more monocyclic aromatics, and reducing carbon deposition on the catalyst. Qian et al.^[^ ^40^ ^]^ (Table 2, entry 28) used Zn/HZSM‐5 for the catalytic pyrolysis of HDPE. Zn/HZSM‐5 was synthesized using two wet synthesis methods, namely incipient wetness impregnation (IWI) and aqueous‐phase ion exchange, followed by H_2_ reduction pretreatment. They investigated the effects of the catalyst synthesis procedure, Si/Al ratio of the zeolite, Zn loading, and type of Zn species on the catalytic pyrolysis of PE pyrolysis vapor. Using 2 wt%‐Zn/HZSM‐5 (IWI) (SiO_2_/Al_2_O_3_ = 25) resulted in a 48% aromatic yield with 93% BTX selectivity based on benzene ring recovery. The maximum aromatic yield (53%) was achieved using 2 wt%Zn/HZSM‐5 (IWI) (SiO_2_/Al_2_O_3_ = 50). They concluded that the main active sites that produced monoaromatics were [ZnOH]^+^ and bridged Zn^2+^ species and that hydrogen pretreatment of the catalysts increased their presence. Chen et al.^[^ ^41^ ^]^ (Table 2, entry 29) employed USY, Fe_2_O_3_/USY, Fe_3_O_4_ + Fe (dominant)/USY, and Fe/USY catalysts to investigate the effect of different iron species on the pyrolysis products of HDPE. The oil yields followed the order: Fe_2_O_3_/USY (72.5 wt%) > Fe_3_O_4_ + Fe (dominant)/USY (69.9 wt%) > Fe/USY (66.7 wt%) > USY (56.5%). The resulting pyrolysis oils were within the gasoline and diesel range, comprising mixtures of C_6–34_ compounds enriched in the C_6–10_ fraction, and consisted predominantly of alkanes. The authors concluded that Fe/USY exhibited higher catalytic activity than the formation of light oil and aromatic compounds.

**Figure 3:**
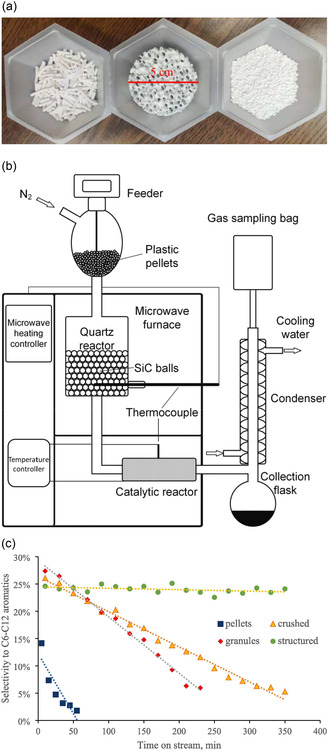
a) Photos of the pelletized catalysts (left), structured catalyst (middle), and granular catalysts (right). b) Schematic diagram of the catalytic pyrolysis system. c) Selectivity of gasoline‐range aromatic hydrocarbons as a function of time on stream for ZSM‐5 catalysts of different geometries at 450 °C and weight hourly space velocity of 40 h^−1^. [Reproduced with permission.^[^ ^42^ ^]^ Copyright 2022, Elsevier (License number: 5955260499507)].

Zhou et al.^[^ ^42^ ^]^ (Table 2, entry 24) studied structured catalysts with ZSM‐5 coated with a 22 μm‐thick film on silicon carbide to improve stability while maintaining catalytic activity (**Figure** **3**a). Silicon carbide is used as a structural material owing to its high thermal conductivity, high mechanical strength, low coefficient of thermal expansion, and chemical inertness at high temperatures. They pyrolyzed waste HDPE using microwave heating at 500 °C, and the pyrolyzates were further reacted with the catalysts at 450 °C (Figure 3b). Compared with pelletized ZSM‐5, the deactivation rate of the structural catalyst for the selectivity of C_6–12_ aromatic hydrocarbons was 1/37 (Figure 3c). In addition, regeneration–recycling tests of the catalyst were conducted to evaluate its stability. The selectivity for aromatic hydrocarbons (C_6–12_) decreased only slightly from 22.5% to 19.4% after five regenerations, indicating excellent stability. Subhashini and Mondal^[^ ^43^ ^]^ (Table 2, entry 30) performed the catalytic pyrolysis of waste plastics using hierarchical ZSM‐5. The small pores in the ZSM‐5 zeolite hindered diffusion when the pyrolysis products were bulky. Hierarchical zeolites have micro‐ and mesopores in their frameworks, which can address this issue and increase their catalytic activity. Compared with pyrolysis, oil yields increased from 21% to 45% for PP, 16% to 53% for HDPE, and 15.1% to 59% for LDPE, ≈2–4 times higher. Furthermore, for PS, the oil yield increased by 9.6%, from 53.4% to 63%, with the highest aromatic content (75%) in the oil compared with those of other plastics.

The effects of combining two different catalysts were also investigated. Uemichi et al.^[^ ^44^ ^]^ (Table 2, entry 4) performed a two‐stage catalytic pyrolysis of PE using SA and HZSM‐5 packed in series in a flow reactor. When SA was used alone, a high yield of oil from gasoline fractional distillation was obtained, but the octane number was low. When HZSM‐5 was used alone, the oil yield was low, and the octane number was high. Therefore, to improve the selectivity for high‐octane gasoline‐fractionated oil, they combined the two catalysts. When SA and HZSM‐5 were used at a weight ratio of 9:1 and temperature of 375 °C, the produced oil consisted solely of the gasoline fraction (C_5–12_), with an oil yield of 58.8 wt%, octane number of 94, and aromatic and benzene contents of 25.2 and 0.9 wt%, respectively. Ratnasari et al.^[^ ^45^ ^]^ (Table 2, entry 12) performed a two‐step catalytic pyrolysis of HDPE by stacking ZSM‐5 (Si/Al = 20) and MCM‐41 (Si/Al = 4) at different weight ratios (ZSM‐5:MCM‐41 = 1:1, 1:3, and 1:7). They combined the two catalysts to catalytically crack the high‐molecular‐weight hydrocarbons produced during the pyrolysis of HDPE into lower‐molecular‐weight hydrocarbons on the MCM‐41 catalyst, allowing them to readily penetrate the microporous structure of the zeolite ZSM‐5 catalyst. The results showed that a catalyst weight ratio of ZSM‐5:MCM‐41 = 1:1 produced an oil yield of more than 80 wt%, with aromatic and gasoline‐range hydrocarbons (C_8–12_) of 95.85 and 97.72 area%, respectively.

Spent fluidized catalytic cracking (FCC) catalysts are inexpensive and effective in cracking polyolefin plastics. Orozco et al.^[^ ^46^ ^]^ studied the catalytic pyrolysis of HDPE in a conical spouted‐bed reactor using spent FCC catalysts (Table 2, entry 20). The conical spouted‐bed reactor facilitated rapid pyrolysis owing to its efficient heat conduction to the plastic and prevented defluidization caused by the viscous polyolefins and waxes (pyrolysis products). They reported reduced wax formation (C_19_<) and the recovery of C_5–11_ aliphatic hydrocarbons. Vollmer et al.^[^ ^47^ ^]^ examined the catalytic pyrolysis of PP using fresh Y‐zeolite and spent FCC catalysts (Table 2, entry 21). The results revealed that metals, such as Fe, Ni, and V accumulated in the spent FCC catalyst, were effective in the decomposition and aromatization of the plastic pyrolyzates.

Sulfated zirconia (SZ) is a very strong acid catalyst suitable for alkylation, esterification, hydrocracking, and various organic synthesis reactions. Almustapha et al.^[^ ^48^ ^]^ (Table 2, entry 11) investigated the catalytic pyrolysis of HDPE in a fixed‐bed reactor using SZ. At 380 °C, the oil yield was 38.5 wt%, and the oil composition consisted of 58% aromatic compounds, 21% olefins, 16% paraffins, and 5% naphthalene. They reported that the carbon number distribution of the oil was mainly within the gasoline range (C_7–13_), with only a small fraction of compounds above C_13_. Additionally, they used sulfated zirconia modified with calcium carbide (SZ/CC) to improve the oil yield (Table 2, entry 16).^[^ ^49^ ^]^ As a result, the oil yield increased to 66 wt%, and the oil composition changed significantly to 74% olefins, 23% naphthenes, 3% paraffins, and less than 0.1% aromatic compounds.

Recent studies have also explored metal oxides such as MgO and Al_2_O_3_ for their catalytic properties. Fan et al.^[^ ^50^ ^]^ (Table 2, entry 14) used MgO for microwave‐assisted pyrolysis of LDPE. Compared to noncatalytic pyrolysis (46.3 wt%), the use of MgO resulted in lower oil yields (24.2–38.5 wt%) but achieved up to 96.0% hydrocarbons in the gasoline range, consisting of 39.7% monocyclic aromatics and 56.3% C_5–12_ aliphatics. The authors reported that the selectivity for monocyclic aromatics increased with higher catalyst‐to‐feedstock ratios, pyrolysis temperatures, and catalytic reaction temperatures. Cai et al.^[^ ^51^ ^]^ (Table 2, entry 18) investigated the catalytic pyrolysis of various plastics using Fe/Al_2_O_3_. The oil yield was about 20 wt%, and the carbon number distribution ranged from C_6_ to C_22_. For PP, the oil was particularly rich in naphthalene (C_10_) at 27.2%, followed by pyrene (C_16_) at 15.7%, anthracene (C_14_) at 15.3%, and biphenyl (C_12_) at 12.7%. The effect of Fe/Al_2_O_3_ ratio on product composition in PP was further examined (Table 2, entry 19).^[^ ^52^ ^]^ At Fe/Al_2_O_3_ ratios of 1:5, 1:2, and 1:1, the composition of the liquid oil shifted significantly, with products consisting primarily of naphthalene (C_10_), anthracene (C_14_), and pyrene (C_16_). Notably, when the Fe/Al ratio was 1:2, the proportion of naphthalene reached a maximum of 71.38 area%. Chen et al.^[^ ^53^ ^]^ (Table 2, entry 26) conducted the pyrolysis of LDPE using various catalysts, including Pt/SiO_2_, Pt/Al_2_O_3_, and Pt‐Fe/Al_2_O_3_. Supporting Pt on Al_2_O_3_ or SiO_2_ significantly increased the oil yield, exceeding 50 wt%. Among these, the Pt‐0.25Fe/Al_2_O_3_ catalyst achieved the highest oil yield of 59.3 wt%, with product selectivities of 29.0% for the gasoline fraction (C_7–12_), 52.1% for the jet fuel fraction (C_9–16_), and 55.2% for the diesel fraction (C_9–22_). Additionally, the oil yield and product selectivity remained stable after catalyst regeneration.

**Figure 4:**
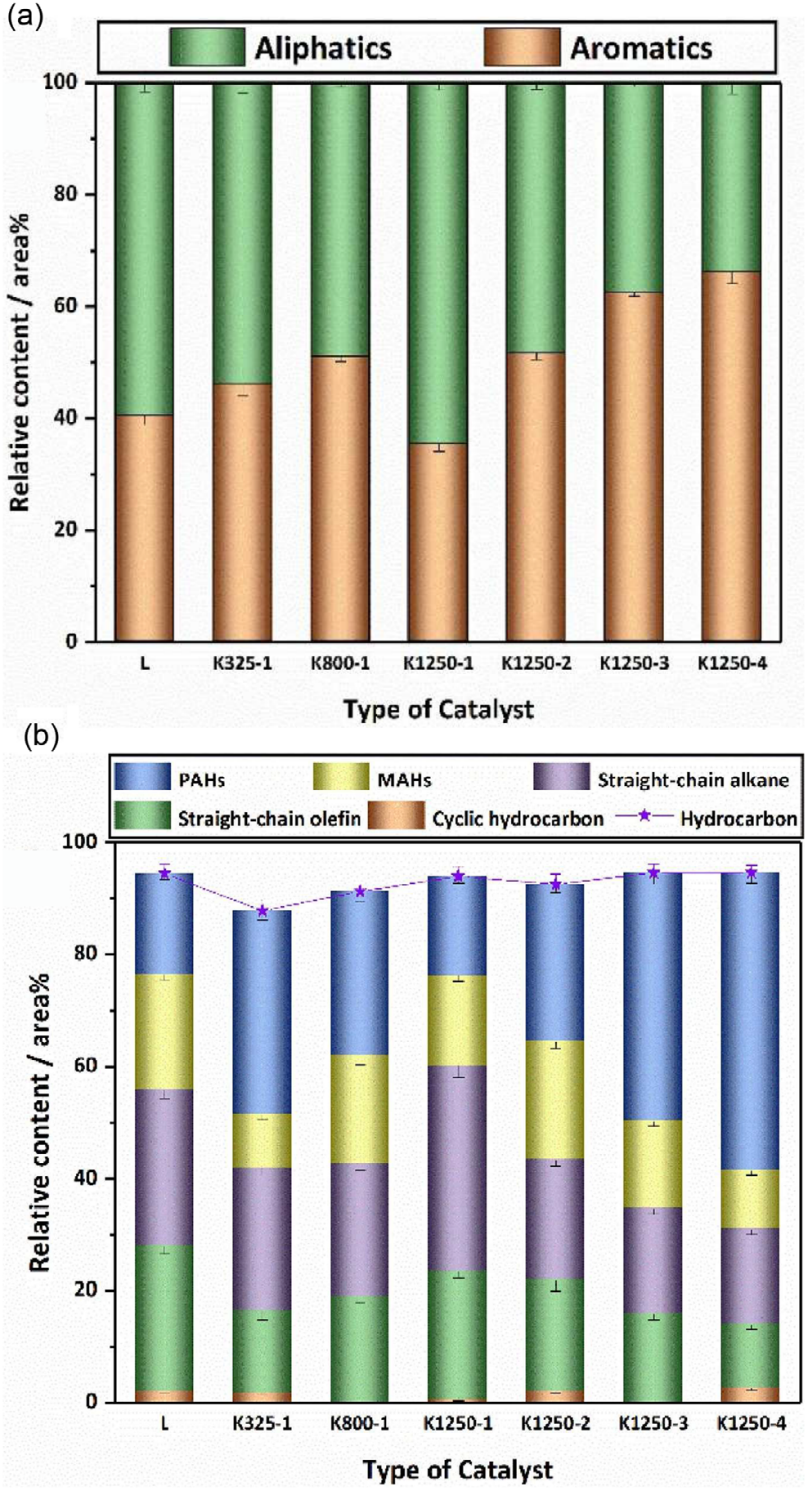
Distribution of oil compounds: a) aromatic and aliphatic content, b) distribution of hydrocarbon compounds. [Reproduced with permission.^[^ ^57^ ^]^ Copyright 2021, Elsevier (License number: 6015091311348)].

Donaj et al.^[^ ^54^ ^]^ (Table 2, entry 9) performed the pyrolysis of mixed plastics using a commercial Ziegler–Natta catalyst (TiCl_4_/MgCl_2_) as the Lewis acid catalyst in a bench‐scale fluidized‐bed reactor (1–3 kg h^−1^). At 650 °C, compared with the noncatalyzed case, the yields of gas products increased from 36.9 to 54.3 wt%, with higher yields of ethylene (22.3 wt%), propylene (21.1 wt%), and methane (21.0 wt%). Li et al.^[^ ^55^ ^]^ (Table 2, entry 13) pyrolyzed mixed plastics containing PET at 500 °C using transition metal (Fe, Ti, Zr, and Al)‐modified pillared clay (PILC) as a catalyst with moderate acidity. The Fe‐PILC demonstrated the best performance, producing 79.3 wt% oil, of which 80.5% was in the diesel fraction (C_13–19_). It also showed the highest H_2_ yield of gaseous products, with a 47.7 vol% yield. In the catalyst regeneration–recycling evaluation, the oil yield of the Fe‐PILC after two regeneration cycles was slightly lower at 75.9 wt%. In addition, no adverse effects on the catalyst were observed owing to the presence of PET‐derived oxygen compounds (such as benzoic acid) in the mixed plastic, and the selectivity of the oil product was maintained after catalyst regeneration. Ding et al.^[^ ^56^ ^]^ (Table 2, entry 15) used NiO (in situ catalyst) and HY zeolite (ex situ catalyst) for the microwave‐assisted pyrolysis of LDPE. The optimal oil yield (56.5 wt%) and oil quality were achieved at a pyrolysis temperature of 500 °C and catalyst temperature of 450 °C. The addition of NiO was effective in obtaining high‐octane‐number oils. Luo et al.^[^ ^57^ ^]^ (Table 2, entry 22) investigated the catalytic pyrolysis of LDPE in a fixed‐bed reactor using kaolin, an abundant and inexpensive material. The oil yield remained around 70 wt% regardless of the kaolin mesh size. Notably, kaolin (1250 mesh) showed an increase in the content of aromatic hydrocarbons—particularly polycyclic aromatic hydrocarbons—in the oil with repeated use (**Figure** **4**a,b). The authors concluded that the increase in particle size and acidity due to progressive coking of the catalyst led to enhanced aromatization. Zhao et al.^[^ ^58^ ^]^ (Table 2, entry 32) used bifunctional ZnO for microwave‐assisted catalytic pyrolysis of various waste plastics. Catalyst stability was evaluated through 50 reuse cycles under the conditions of 280 °C reaction temperature, 30 min reaction time, and a ZnO:wPP = 10 g:50 g. The oil yield was consistently maintained at ≈80 wt%, indicating high catalyst stability. The carbon number distribution of the oil ranged from C_8_ to C_40_, with a viscosity index (VI) of 122.2 and a pour point of 41.2 °C.

## 3 Latest Trends in Industrial‐Scale Pyrolytic Liquefaction Projects in Japan, Europe, and the US

After a series of studies on the conversion of waste plastics into chemical raw materials through pyrolysis began in the 1970s, several pilot‐scale chemical recycling projects were launched in Europe and the US during the 1990s. However, these projects were short‐lived, lasting only a few years because of cost issues or a lack of waste collection.^[^ ^59^ ^]^ In Japan, alongside the enforcement of the Containers and Packaging Recycling Law in 1997, several liquefaction plants for packaging plastic were established in cities such as Niigata (6000 t year^−1^) and Sapporo (15,000 t year^−1^).^[^ ^60^ ^]^ These plants began commercial operations in 1999 and 2000 and continued stable operation for ≈10 year with the support of the law. However, because of the Japanese government's policy of prioritizing mechanical recycling over chemical recycling, these plants were forced to close in 2007 and 2009 because of insufficient waste collection. Currently, chemical recycling methods in Japan include chemical raw materials for coke ovens, reducing agents in blast furnaces by steel manufacturers, and the production of synthetic gas by chemical companies.^[^ ^14^ ^]^ However, the global demand for plastic recycling has increased dramatically over the past few years, and pyrolysis methods that can process a wider variety and volume of plastics than material recycling are attracting increasing attention. Therefore, various companies operate pyrolysis liquefaction plants or conduct pilot‐ or industrial‐scale demonstration tests on the pyrolysis liquefaction of waste plastics.

**Figure 5:**
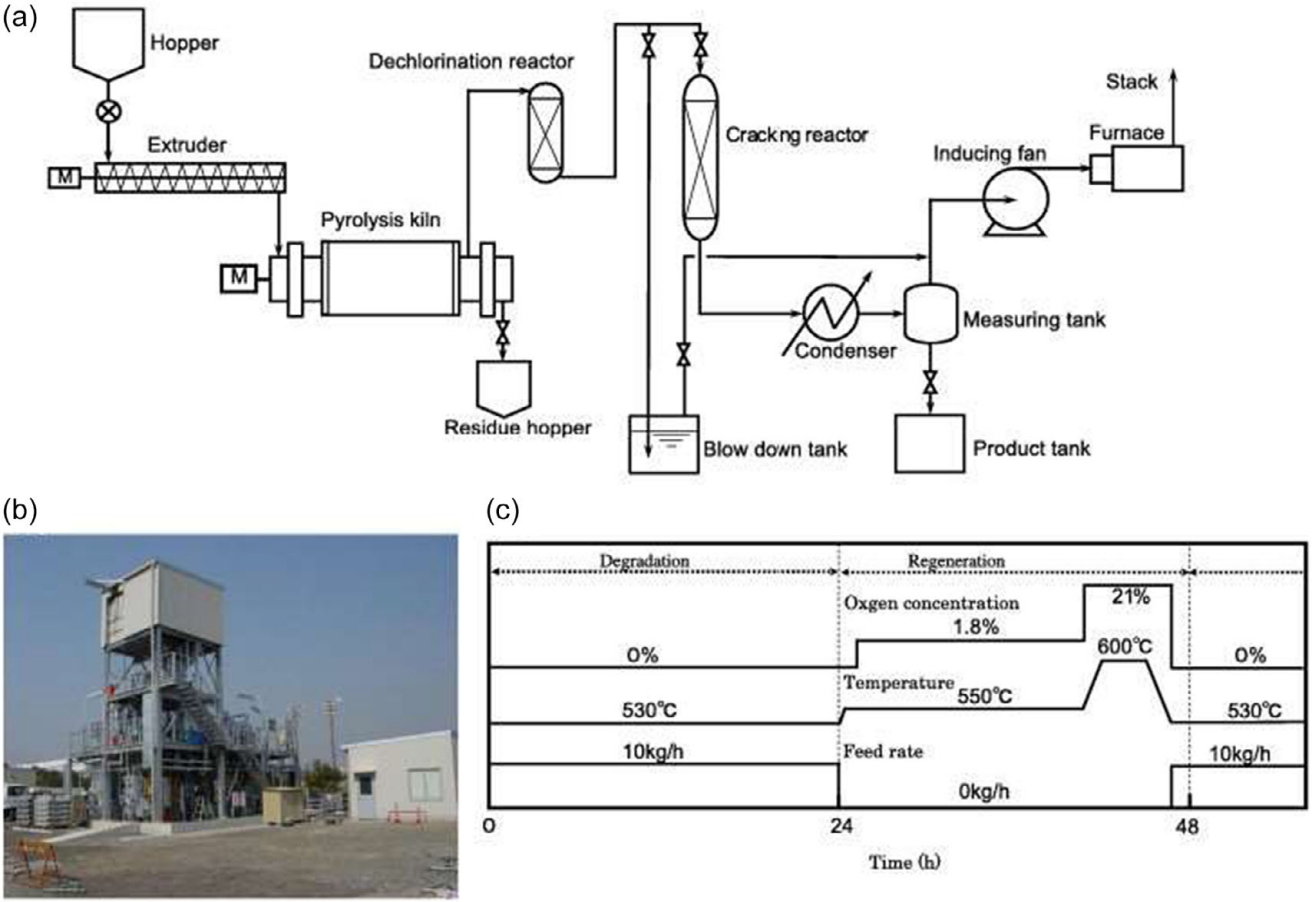
General features of the pilot plant (10 kg h^−1^): a) Flow diagram of the pilot plant, b) appearance of the pilot plant, and c) operating conditions for the cracking reactor. [Reproduced with permission.^[^ ^62^ ^]^ Copyright 2008, Elsevier (License number: 5955261115154)].

**Figure 6:**
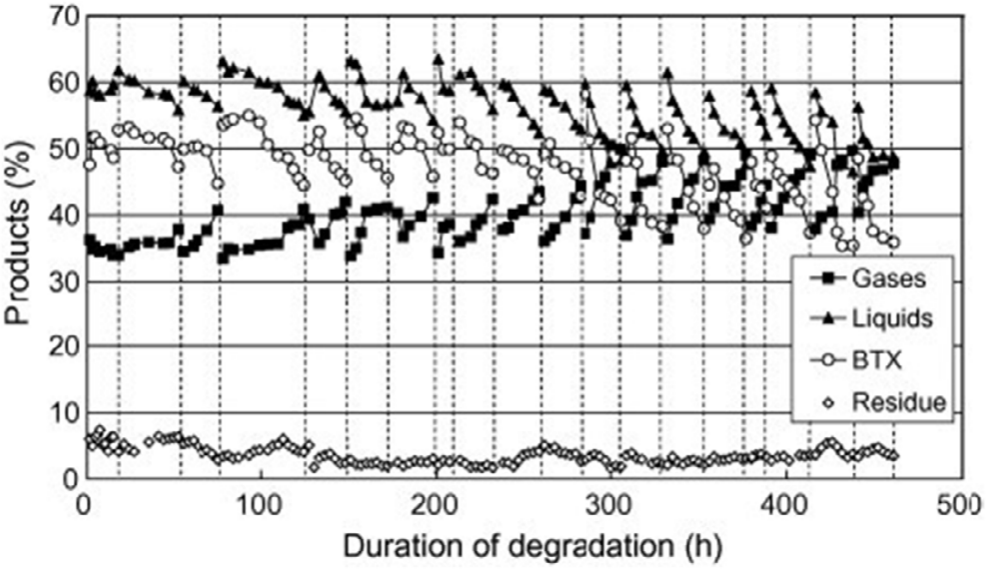
Relationship between product distribution and degradation time for industrial plastic waste: Cracking temperature of 524–543 °C, with a weight/flow ratio of 90 g min g^−1^. [Reproduced with permission.^[^ ^62^ ^]^ Copyright 2008, Elsevier (License number: 5955261115154)].

Mertinkat et al.^[^ ^61^ ^]^ investigated the pyrolysis of LDPE and PS at a feeding rate of ≈1 kg h^−1^ using a spent FCC catalyst as the fluidized bed material. The catalytic pyrolysis of PS significantly altered the product distribution, producing ethylbenzene (18–26 wt%), benzene (9–22 wt%), styrene (1–7 wt%), and toluene (3–5 wt%), compared with that of styrene (61 wt%) produced without catalyst. For PE, even at low temperatures (450–515 °C), gas (48–52 wt%) and oil (38–39 wt%) consisting of low‐boiling‐point aliphatic hydrocarbons were obtained, replacing the wax (up to 90 wt%) produced under non‐catalytic conditions. Nishino et al.^[^ ^62^ ^]^ conducted the catalytic pyrolysis of industrial plastic waste (IPW), which consisted of at least 96 wt% PE, containing 300 ppm Cl and less than 100 ppm Br, and plastic waste collected under the Containers and Packaging Recycling Law (RLW), which contained 60 wt% PE and 34 wt% PP, with 1300 ppm Cl and less than 50 ppm Br. The pyrolysis was conducted in a pilot plant with a maximum feeding rate of 10 kg h^−1^ (**Figure** **5**). Ga‐ZSM‐5 (Si/Ga = 35) was used for continuous operation under alternating pyrolysis and catalyst regeneration. The oil yield from IPW exceeded 50%, with 80% more than 80% of the yield consisting of aromatic compounds. Furthermore, over 90% of the aromatic compounds were BTX (**Figure** **6**). The process was operated continuously for 460 h using IPW as the feedstock. By contrast, when RLW was used as the feedstock, the process could only operate continuously for 170 h because of residue deposition on the inner reactor walls. The oils obtained from IPW and RLW had high Cl contents, with inorganic Cl concentrations of 2.2 μg g^−1^ for IPW and 19 μg g^−1^ for RLW, and organic Cl concentrations of 6.6 μg g^−1^ for IPW and 25 μg g^−1^ for RLW. Therefore, the authors concluded that the dechlorination pretreatment of Cl‐containing plastics is necessary.

**Table** **3** summarizes the operating industrial‐scale projects that produce plastic pyrolysis oil or utilize pyrolysis oil in Japan, Europe, and the US based on press‐released information. The CFP Group^[^ ^63^ ^]^ (Table 3, entry 3) has successfully produced pyrolysis oil that does not solidify at temperatures below 0 ºC by using a waste plastic‐to‐oil conversion plant. This plant can process PVC‐containing plastics (up to 10%), ethylene‐vinyl acetate, ethylene‐vinyl alcohol copolymers, and N‐ and S‐containing plastics. In addition, the off‐gases generated during pyrolysis, including combustible gases such as methane, ethane, propane, and butane, are recycled as fuel for the plant. This facility has been installed in Okayama (Japan), Johor (Malaysia), and Cape Town (South Africa). Mitsui Chemicals Inc.^[^ ^64^ ^]^ (Table 3, entry 4) introduced plastic pyrolysis oil delivered from the CFP group into a naphtha cracker to produce chemical feedstocks such as ethylene, propylene, C_4_ and C_5_ fractions, and benzene. Mitsui Chemicals Inc. has obtained International Sustainability and Carbon Certification (ISCC) PLUS certification (a sustainability certification for the circular economy and bioeconomy offered by the ISCC based on the use of alternative feedstocks)^[^ ^65^ ^]^ and plans to market chemical recycling products using the mass balance method. Environment Energy Co. Ltd.^[^ ^66^ ^]^ (Table 3, entry 1) demonstrated the pyrolytic liquefaction of waste plastics using HiCOP technology,^[^ ^67^ ^]^ which is a process for obtaining oil from waste plastics using a spent FCC catalyst. This plant produces wax‐free oil in a high yield (80%). In addition, this process reduces the Cl content in the oil using calcium hydroxide (Ca(OH)_2_) as a Cl absorber. Environmental Energy Co. Ltd. and Idemitsu Kosan Co. Ltd. plan to introduce the pyrolysis oil produced by this technology into oil refinery processes to produce petrochemical feedstocks.^[^ ^68^ ^]^ Therefore, they established a joint venture company, Chemical Recycle Japan Co. Ltd., to start commercial operations by 2025 (20,000 t year^−1^).^[^ ^69^ ^]^ ENEOS Co.^[^ ^70^ ^]^ and Mitsubishi Chemical Co.^[^ ^71^ ^]^ (Table 3, entry 5) plan to operate a joint project for the liquefaction of waste plastics through the use of Mura Technology's hydrothermal technology. The obtained oil was used as feedstock in an existing chemical plant to produce basic chemicals such as ethylene, propylene, benzene, and their derivatives. Mitsubishi Chemical Co. also obtained the ISCC PLUS certification. Denka Company Limited^[^ ^72^ ^]^ and Toyo Styrene Co. Ltd. (an equity method affiliate)^[^ ^73^ ^]^ (Table 3, entry 2) completed the construction of a chemical recycling plant for PS at the DENKA Chiba Plant (Ichihara, Japan). The plant uses technology^[^ ^74^ ^]^ licensed from Agilyx (US), which is a catalyst‐free pyrolysis process capable of processing a wide range of PS materials and their blends. Toyo Styrene Co. Ltd. used recycled styrene monomers produced by this process to provide ISCC PLUS‐certified styrene.

**Table 3:** Press‐Released industrial‐scale projects for pyrolytic liquefaction of waste plastics and utilization of pyrolysis oil in Japan, Europe, and the US.

Entry	Plant location	Company	Materials	Technology	Target products or product name and their respective yields (if available)	Current plastic feed scale (including the expected amount)	References
1	Japan	Environment Energy Co., Ltd.	Waste plastics	Catalytic conversion over spent FCC catalyst (HiCOP technology)	A mixture of ≈50% gasoline (naphtha) and 50% diesel (light oil)	120 t mo^−1^ (200 kg h^−1^)	Environment Energy Co. Ltd.^[^ ^66^ ^]^
2	Chiba (Japan)	Denka Company Limited and Toyo Styrene Co., Ltd.	Wide range of polystyrene raw materials and blends	Agilyx's catalyst‐free pyrolysis technology	Styrene monomer	10 t d^−1^ (3000 t year^−1^)	Denka Company Limited^[^ ^72^ ^]^
3	Okayama (Japan)	CFP Group	Waste plastics (mainly PE, PP, and PS)	Production of pyrolysis oil with no solidification observed below 0 °C	Light, medium, and heavy oils	9000 t year^−1^ (30 t d^−1^)	CFP Group^[^ ^63^ ^]^
4	Osaka (Japan)	Mitsui Chemicals, Inc.	Plastic pyrolysis oil	Introduction of pyrolysis oil into a naphtha cracker	C_2__–__5_ aliphatic hydrocarbons, benzene, and others	Not reported	Mitsui Chemicals, Inc.^[^ ^64^ ^]^
5	Ibaraki (Japan)	ENEOS Co. and Mitsubishi Chemical Co.	Waste plastics	Mura Technology's supercritical water technology	Naphtha fraction	20,000 t year^−1^	ENEOS Co.^[^ ^70^ ^]^ and Mitsubishi Chemical Co.^[^ ^71^ ^]^
6	Ennigerloh (Germany)	CARBOLIQ GmbH	Mixed packaging plastics (70%) and celluloses (30%)	Catalytic tribochemical conversion	CARBOLIQ‐CLR: liquid mixture of hydrocarbons	600–1000 kg h^−1^	CARBOLIQ GmbH^[^ ^78^ ^]^
7	Seville, Almeria (Spain)	Plastic Energy	Waste plastics (LDPE, HDPE, PP, and PS)	Pyrolysis	Thermal anaerobic conversion oil	7000 t year^−1^	Plastic Energy^[^ ^83^ ^]^
8	Schwechat (Austria)	OMV	Waste plastics	Pyrolysis	Shorter‐chain light hydrocarbons	Total of 200,000 kg from 2018 to May 2019	OMV^[^ ^84^ ^]^
9	Oostende (Belgium)	BlueAlp	Waste plastics	Catalyst‐free slow‐cracking process	Pyrolysis oil	17,000 t year^−1^	BlueAlp^[^ ^85^ ^]^
10	Skive (Denmark)	Quantafuel	Waste plastics	Catalytic hydrogenation	10% Gas, 10% char, and 80% oil (16% light fraction (C_6–12_), 56% diesel fraction (C_11–21_), and 8% heavy fraction (C_20–28_))	20,000 t year^−1^	Quantafuel^[^ ^76^ ^]^
11	Teesside (UK)	Mura Tecnology	Waste plastics (LDPE, HDPE, PP, and PS)	Supercritical water technology	Naphtha, light oil, heavy oil, and wax	Recycled liquid hydrocarbon production volume of 20,000 t year^−1^	Mura Tecnology^[^ ^81^ ^]^
12	Rotterdam (Netherland)	Pryme	Waste plastics	Pyrolysis	Pyrolysis oil (most of the identified compounds are in the light‐ and middle‐distillate ranges)	40,000 t year^−1^	Pryme^[^ ^89^ ^]^
13	Andalucia (Spain)	Honeywell	Waste plastics (mainly PE, PP, and PS; PVC can be processed up to 4%)	Pyrolysis	20%–35% Naphtha, 45%–60% distillate, 2%–10% gas oil, <500 ppm S, <2000 ppm N, <15 ppm chloride, and 25%–50% olefins	Not reported (expected to be 30,000 t year^−1^)	Honeywell^[^ ^87^ ^]^
14	Wesseling (Germany)	LyondellBasell	Waste plastics (mixed plastic packaging and flexible polyolefin materials)	Catalytic pyrolysis	Pyrolysis oil and gas (used as polymer feedstock and fuel)	Not reported (expected to be 50,000 t year^−1^)	LyondellBasell^[^ ^80^ ^]^
15	Texas (US)	Exxon Mobil	Waste plastics	Pyrolysis	Chemical feedstock	40,000 t year^−1^	Exxon Mobil^[^ ^91^ ^]^
16	Indiana, Georgia (US)	Brightmark	Waste plastics (including PET and PVC processing)	Pyrolysis	Ultra‐low sulfur diesel, wax, and naphtha	100,000 t year^−1^	Brightmark^[^ ^92^ ^]^
17	Texas (US)	New hope energy	Waste plastics (mainly PE, PP, and PS, with PVC and nylon limited to minimum amounts)	Pyrolysis	Hydrocarbons for further processing: 70% Asphalt‐like material: 5% Lighter gas for fuel to heat the system: 25%	100,000 t year^−1^^[^ ^95^ ^]^	New Hope Energy^[^ ^93^ ^]^
18	Texas (US)	Anellotech	Waste plastics (including PET and PVC processing)	Fluidized‐bed catalytic pyrolysis	Light olefins, aromatic compounds, and paraffins	0.5 t d^−1^ (demo plant)	Anellotech^[^ ^97^ ^]^
19	Ohio (US)	Alterra Energy	Waste plastics	Pyrolysis	Small‐chain hydrocarbons	60 t d^−1^	Alterra Energy^[^ ^98^ ^]^
20	US	Plastic2Oil	Waste plastics	Catalytic pyrolysis	≈1 gal of fuel is extracted from 8.3 lbs of plastic	4000 lb h^−1^	Plastic2Oil^[^ ^99^ ^]^

BASF launched the ChemCycling project to enhance the industrial‐scale chemical recycling of waste plastics and cooperate with technology partners to produce pyrolysis oil.^[^ ^75^ ^]^ Quantafuel^[^ ^76^ ^]^ (Table 3, entry 10) conducted the catalytic pyrolysis of mixed plastic wastes using hydrogenation catalysts (20,000 t of plastic per year), which were supported by BASF.^[^ ^77^ ^]^ CARBOLIQ GmbH^[^ ^78^ ^]^ (Table 3, entry 6) demonstrated a one‐step catalytic pyrolysis process (catalytic tribochemical conversion (CTC)). According to this information, the plant accepts packaging plastics, cellulose, PVC, and acrylonitrile‐butadiene‐styrene (ABS).^[^ ^79^ ^]^ LyondellBasell^[^ ^80^ ^]^ (Table 3, entry 14) is operating a catalytic pyrolysis process. A new plant in Wesseling (Germany) is set to process 50,000 t of plastic waste annually. Mura Technology^[^ ^81^ ^]^ (Table 3, entry 11) opened the world's first commercial‐scale hydrothermal plastic recycling technology (Hydro‐PRT) advanced plastic recycling plant. Mura Technology developed a supercritical hydrothermal pyrolysis technology by employing Licella's catalytic hydrothermal reactor,^[^ ^82^ ^]^ which can convert more than 85 wt% of plastics into hydrocarbons. This plant produces 20,000 t of hydrocarbons per year. Plastic Energy^[^ ^83^ ^]^ (Table 3, entry 7) conducts chemical recycling of waste plastics using pyrolysis technology. The plant has a processing capacity of 7000 t year^−1^, and the pyrolysis oil has been commercialized. OMV^[^ ^84^ ^]^ (Table 3, entry 8) is developing a recycling technology in which waste plastics are heated to 400–450 °C to produce pyrolysis oil. The pilot plant was operated at the Schwechat (Austria) refinery in 2018, accumulating over 27,000 h of operation, with 200,000 kg of plastics processed between 2018 and 2019. BlueAlp^[^ ^85^ ^]^ (Table 3, entry 9) has operated a pilot‐scale pyrolysis plant with a processing capacity of 3000 t year^−1^ since 2014. Since 2020, the company has run a commercial‐scale plant with a capacity of 17,000 t of plastics per year. In addition, the company is currently partnering with Recupero Etico Sostenible to build its first industrial‐scale advanced recycling plant in Italy, with a processing capacity of 20,000 t year^−1^, slated for operation in mid‐2026.^[^ ^86^ ^]^ Honeywell^[^ ^87^ ^]^ (Table 3, entry 13) announced a joint venture with Sacyr to build a plastic waste recycling plant in Andalusia, Spain, with a processing capacity of 30,000 t of waste plastics per year. The plant will be equipped with Honeywell's pyrolysis technology. Honeywell signed a strategic agreement in 2022 with TotalEnergies to promote the development of advanced plastic recycling.^[^ ^88^ ^]^ Pryme^[^ ^89^ ^]^ (Table 3, entry 12) operated a plastic waste pyrolysis plant with a processing capacity of 40,000 t year^−1^ in Rotterdam (Netherlands). Shell has signed strategic cooperation agreements with various partners, and an upgrading plant for pyrolysis oil is currently under construction in the Netherlands.^[^ ^90^ ^]^ The plant is designed to process up to 50,000 t of pyrolysis oil per year.

Exxon Mobil^[^ ^91^ ^]^ (Table 3, entry 15) announced the start‐up of advanced recycling facilities in North America (Baytown, Texas) in December 2022. ExxonMobil's technology can process 40,000 t of plastic waste per year. In addition, ExxonMobil is progressing with plans to achieve an advanced recycling capacity of up to 500,000 t year^−1^ by 2027 at multiple sites worldwide, including the U.S. Gulf Coast, Canada, the Netherlands, and Singapore. Brightmark^[^ ^92^ ^]^ (Table 3, entry 16) employs a technology capable of converting waste plastics, including PET and PVC, into diesel, wax, and naphtha with an ultralow S content. In addition, Brightmark announced plans to build an advanced plastic reclamation facility at New South Wales, Australia. New Hope Energy^[^ ^93^ ^]^ (Table 3, entry 17) has collaborated with Lummus Technology^[^ ^94^ ^]^ to develop a pyrolysis technology. The company is conducting a demonstration study on the chemical recycling of waste plastics at its plant in Texas, US, which has a processing capacity of 100,000 t of waste plastics per year.^[^ ^95^ ^]^ In addition, New Hope Energy partnered with Total Energies in 2022.^[^ ^96^ ^]^ At its Texas plant, New Hope Energy converts waste plastics into recycled feedstock, a portion of which is purchased and converted into a virgin‐quality polymer suitable for food packaging. Anellotech^[^ ^97^ ^]^ (Table 3, entry 18) has developed a catalytic pyrolysis technology for converting plastic waste into light olefins, aromatic compounds, and paraffins. Utilizing a fluidized‐bed catalytic process, this technology can handle waste plastics such as PVC and PET. Currently, Anellotech is demonstrating this technology at its demo plant in Texas. The commercial plant will be able to process more than 200,000 tons year^−1^ of plastic waste. Alterra Energy^[^ ^98^ ^]^ (Table 3, entry 19) and Plastic2Oil^[^ ^99^ ^]^ (Table 3, entry 20) have also developed pyrolysis oil conversion technologies used in the US.

## 4 Latest Trends in Expanding the Range of Acceptable Plastics in Pyrolysis Plants

As indicated in Section 3, commercial‐scale plants have attempted to feed pyrolysis products back into existing petroleum‐refining facilities to produce petrochemicals. In addition, some plants are developing technologies to convert waste plastics, including PVC, into chemical feedstocks. The allowable Cl concentration in the fossil‐based steam cracker feedstock has been reported to be 3 ppm.^[^ ^60^ ^]^ In Japan, the “Act on the Promotion of Sorted Garbage Collection and Recycling of Containers and Packaging” specifies that the Cl content in pyrolysis oil must be less than 100 ppm.^[^ ^100^, ^101^ ^]^ As of 2023, PVC accounts for 12.8% of global plastic production.^[^ ^102^ ^]^ The generation of WEEE and Br‐containing plastic waste has increased. Therefore, the effective processing of halogen‐containing plastics, such as PVC and BFR‐containing plastics, is critical for advancing commercial‐scale pyrolysis technologies for chemical feedstock production. In addition, the pyrolysis of PET mainly produces terephthalic acid (TPA), a high‐boiling‐point acidic compound that causes corrosion and pipe blockages in treatment facilities.^[^ ^103^, ^104^ ^]^ Thus, PET is unsuitable for pyrolysis. Therefore, developing methods for recovering commercial chemical feedstocks from these plastics is essential for improving the global plastic recycling rate.

### 4.1 Halogen Removal from Pyrolysis Products

Research trends in the catalytic pyrolysis of halogen‐containing plastics are summarized in **Table** **4**. In the catalytic pyrolysis of PVC, several studies have reported the use of various alkaline additives (CaO, Ca(OH)_2_, CaCO_3_, NaHCO_3_, and Na_2_CO_3_) and transition metal oxides (TiO_2_, V_2_O_5_, MoO_3_, MnO_2_, Fe_2_O_3_, CuO, ZnO, NiO, and La_2_O_3_) for the removal of HCl. Fekhar et al.^[^ ^105^, ^106^ ^]^ (Table 4, entries 6 and 7) performed the pyrolysis of waste mixed plastics using catalysts such as Ni/ZSM‐5, Ni/SAPO‐11, Ca(OH)_2_, and red mud. At a pyrolysis temperature of 550–560 °C without a catalyst, the yields of heavy oil, light oil, and gas were 49.7%, 36.8%, and 8.6%, respectively. The use of catalysts increased the yields of gas and light oil to 14.1–21.7% and 49.1–59.7%, respectively. The Cl content in diesel oil was reduced from 0.6% to 0.03–0.26%. Furthermore, the Ni/SAPO‐11 catalyst showed excellent dechlorination performance, reducing the Cl concentration in the pyrolysis oil from 4364 to 228 ppm (94.8% Cl removal). The presence of red mud and Ca(OH)_2_ in the catalyst mixture significantly reduced the Cl content in the pyrolysis oil.

**Table 4:** Summary of the pyrolytic approach to remove halogen from pyrolysis products.

Entry	Year	Plastics (values: wt%)	Catalyst or absorbent	Experimental conditions	Key result	Halogen behavior	References
1	2003	MPa) (30 PE, 30 PP, 30 PS, and 10 PVC)	Ca–C composite	Two‐stage FxBRr); pyrolysis: 430 °C; catalytic upgrading: 350 °C.	67 wt% oil and 24 wt% gas obtained with a Ca–C catalyst.	The Cl concentration in the pyrolysis oil was 360 ppm. The catalyst completely removed Cl from the oil.	Bhaskar et al.^[^ ^107^ ^]^
2	2012	HIPSb)	CaO, Ca(OH)_2_, and oyster shells	FBRs); Catalysis inputs of 40 and 80 g; feed rates of 4.4 and 4.6 g min^−1^; target temperatures ranging from 422 to 460 °C.	Pyrolysis oil primarily consisted of styrene, ethylbenzene, cumene, and other aromatic hydrocarbons. Ca‐based catalysts increased the styrene concentration while reducing the ethylbenzene and cumene concentrations.	The total Br content in the pyrolysis oil was ≈5 wt%, decreasing to 1.3 wt% with Ca(OH)_2_ and 2.7 wt% with oyster shells.	Jung et al.^[^ ^113^ ^]^
3	2015	Cu‐coated PCBc)	Ca(OH)_2_	FxBR; mass ratio of PCB to Ca(OH)_2_ was 1:1; target temperature set to 800 °C at a heating rate of 5 °C min^−1^, with an isothermal temperature held at 700 °C.	Ca(OH)_2_ promoted the formation of phenolic compounds from the PCB matrix mainly at temperatures below 300 °C, while fixation of brominated compounds occurred predominantly at temperatures above 300 °C.	Ca(OH)_2_ removed up to 94% of HBr and 98% of brominated phenols from the pyrolysis products.	Kumagai et al.^[^ ^114^ ^]^
4	2017	HIPS	Fe/ZSM‐5, Fe/MCMl)‐41, Ni/ZSM‐5, and Ni/MCM‐41	Two‐stage FxBR; mass ratio of HIPS to catalyst was 5:0.5; pyrolysis: 450 °C; catalytic upgrading: 450 °C.	Compared with pyrolysis alone (69.0 wt%), oil yields decreased to 63.2 wt% for Fe/ZSM‐5 and 61.2 wt% for Ni/ZSM‐5, while Fe/MCM‐41 and Ni/MCM‐41 showed comparable yields of 65.9 and 65.3 wt%, respectively. The Fe‐modified catalyst increased the yield of monocyclic aromatics, whereas the Ni‐modified catalyst increased the formation of bicyclic aromatics.	The Fe‐modified catalysts captured more inorganic Br than the Ni‐modified catalyst and were advantageous in removing Br from the oil.	Ma et al.^[^ ^118^ ^]^
5	2018	PCB	Fe‐ and Ni‐metal powders (100–150 μm)	Two‐stage FxBR; mass ratio of PCBs to catalyst was 1:1; pyrolysis: 500 °C; catalysis upgrading: 500 and 600 °C.	The maximum oil yield of 50.1 wt% was obtained at 500 °C without catalysts. Ni decreased the oil yield to 29.1 wt% at 600 °C and increased the gas yield. The presence of metal particles promoted the formation of aromatic compounds such as phenol, benzene, and toluene.	Adding Fe and Ni particles achieved a higher debromination efficiency, with the Br content in the oil being less than 2.9%.	Ma and Kamo^[^ ^119^ ^]^
6	2019	wd) MP (35 LDPE, 32 HDPE, 24 PP, 4 PVC, 3 ethylene‐propylene dimers, and 2 PS)	Mixture of Ni/ZSM‐5 (Si/Al = 15.1), Ca(OH)_2_, and RMm); mixture of Ni/SAPOn)‐11 (Si/Al = 0.25), Ca(OH)_2_, and RM	FxBR; target temperatures ranging from 510 to 520 °C.	The aromatics content of 1.9% without a catalyst increased to 11.4–17.6% with Ni/ZSM‐5 and to 8.1–11.0% with Ni/SAPO‐11.	The Ni/SAPO‐11 catalyst reduced the Cl concentration in the pyrolysis oil from 4364 to 228 ppm.	Fekhar et al.^[^ ^105^ ^]^
7	2019	wMP (35 LDPE, 32 HDPE, 24 PP, 4 PVC, and 5 others)	Mixture of Ni/ZSM‐5(Si/Al = 15.1), Ca(OH)_2_, and RM; mixture of Ni/SAPO11 (Si/Al = 0.25), Ca(OH)_2_, and RM	FxBR; Target temperatures ranging from 550 to 560 °C.	The gas yield of 8.6% in the absence of a catalyst increased to 14.1–21.7% with the catalysts. The light oil yield of 36.8% without the catalyst increased to 49.1–59.7% with the catalysts.	The Cl content in gas and light oil was reduced from 9.51% to 4.07–8.53% and from 0.6% to 0.03–0.26%, respectively.	Fekhar et al.^[^ ^106^ ^]^
8	2021	WPCBe)	K_2_CO_3_, Na_2_CO_3_, NaOH, ZSM‐5, and kaolin	Microwave‐assisted FxBR; mass ratio of WPCBs to additive was 5:1; target temperature set to 550 °C.	The oil obtained at 550 °C contained 12.57% MAHt) s (excluding phenol) and 17.7% C_11–20_ aliphatic hydrocarbons. With the addition of ZSM‐5, MAHs increased to 17.02%, and C_11–20_ hydrocarbons increased to 29.45%.	Br fixation increased from 29.11% to 99.80%, 96.39%, and 86.69% with K_2_CO_3_, Na_2_CO_3_, and NaOH, respectively.	Zhang et al.^[^ ^115^ ^]^
9	2021	PC‐based TBBPAf)	Cu_2_O	FxBR; mass ratio of PC‐based TBBPA to Cu_2_O was 3.8:1; targeted temperatures were 390, 480, and 600 °C, with a heating rate of 10 °C min^−1^.	In the presence of Cu_2_O, gas and oil production decreased, while char production increased. The oil was rich in valuable chemicals, including phenols, carbonyls, ethers, and aromatics, but also contained brominated organic compounds.	Cu_2_O significantly reduced the brominated organic compounds in the oil from 6–7 to 0.2 (480 °C) and 0.9 wt% (600 °C). In addition, 99% of HBr was removed with Cu_2_O at 600 °C.	Oleszek et al.^[^ ^116^ ^]^
10	2022	wMP (LDPE, PP, and PVC)	CaO	Mass ratio of feedstock to CaO was 1:1 (FxBR) and 200 g h^−1^:40 g (FBR); thermal pretreatment in auger reactor was conducted at 300 °C; pyrolysis or catalytic pyrolysis in the FxBR was conducted at 500 °C with a heating rate of 20 °C min^−1^; a CaO‐filled hot filter was maintained at 400 °C; two‐stage pyrolysis process: auger reactor + fluidized‐bed reactor + CaO‐filled hot filter.	The two‐stage pyrolysis produced an oil rich in monoaromatics, with 80 wt% oil yield. Benzene was the main component, comprising 55 wt% of the oil.	Thermal pretreatment yielded 90.4% residue, decreasing the total Cl content from 8100 to 2800 ppm (65.4% removal rate). A two‐stage treatment further reduced the Cl concentration in the oil to 68 ppm.	Park et al.^[^ ^108^ ^]^
11	2022	PVC‐containing WEEE plastics	ZSM‐5 (Si/Al = 11), ZSM‐5 (Si/Al = 25), and desilicated ZSM‐5 (Si/Al = 25)	Thermal pretreatment in a downdraft reactor at 350 °C with a heating rate of 10 °C min^−1^ for 30 min; two‐stage downdraft FxBR reactor; pyrolysis: 600 °C; catalytic upgrading: 450 °C.	Ex situ desilicated ZSM‐5 produced the highest oil yield of ≈60 wt% with over 50 wt% MAHs in the oil.	The thermal pretreatment of e‐waste plastic removed 87% of Cl as HCl, with the lowest Cl concentration of 88 ppm achieved using desilicated ZSM‐5.	Marino et al.^[^ ^109^ ^]^
12	2022	MP (ABSg), HIPS, PC, and PP)	ZSM‐5 (containing 30 wt% crystalline zeolite on an SAo) matrix), Al_2_O_3_, MgO, Fe/Al_2_O_3_, and Fe/MgO	Pyrolysis‐gas chromatography/MS and FxBR; mass ratio of MP to catalyst was 2:1; target temperature was 440 °C.	The catalysts promoted the formation of phenolic compounds, with Fe/Al_2_O_3_ significantly enhancing their production. Fe/Al_2_O_3_ was the most effective catalyst for increasing phenolic compounds and facilitating debromination.	The debromination rate followed this order: Fe/Al_2_O_3_ > Fe/MgO > MgO > ZSM‐5 > Al_2_O_3_.	Charitopoulou et al.^[^ ^117^ ^]^
13	2022	MP (35 LDPE, 25 PP, 20 HDPE, 10 PS, and 10 PVC)	Ca(OH)_2_‐extrudate, Fe_3_O_4_‐Si, and Fe_3_O_4_‐Si_(reduction)	Two‐stage FxBR; conventional pyrolysis: 500 °C; Stepwise pyrolysis: first step: 350 °C, second step: 500 °C; Catalytic upgrading: 300 °C.	Noncatalyst → Oil: 67.0 ± 0.7 wt%, wax: 29.0 ± 2.7 wt%, gas: 16.9 ± 1.3 wt%; Catalyst → Oil: 64.5–67.2 wt%, wax: 3.9–7.5 wt%, gas: 25.1–26.4 wt%.	The Cl concentration in the pyrolysis oil decreased from 7789 ppm to less than 86 ppm using the catalyst.	Hubacek et al.^[^ ^110^ ^]^
14	2023	MP (PE, PP, PS, and PVC)	Carbon‐based catalyst (Fe/Ni/Mo@N/C) + HZSM‐5 (SiO_2_/Al_2_O_3_ = 25, mol.p) ratio), and MCM‐41 (SiO_2_/Al_2_O_3_ = 25, mol. ratio) + HZSM‐5	Two‐stage FxBR; In situ catalysis: 500 °C; ex situ catalysis: 550 °C.	By combining hydrothermal pretreatment, the BTEXu) yield increased from 64.31% to 71.29% with the Fe/Ni/Mo@N/C+HZSM‐5 catalyst and from 59.35% to 70.41% with the MCM‐41+HZSM‐5 catalyst.	Hydrothermal pretreatment of MP enhanced dechlorination and improved the BTEX selectivity. Fe/Ni/Mo@N/C showed a better Cl resistance than MCM‐41.	Wang et al.^[^ ^111^ ^]^
15	2023	MP (90 PP and 10 PVC)	Ru/TiO_2_, Mg_3_AlO_4.5_	Autoclave reactor; 50–375 °C, 3 MPa, H_2_ or He atmosphere, 6 h; upgrading conditions: 0.1 g Ru/TiO_2_, 250 °C, 3 MPa, 16 h, H_2_ atmosphere.	The two‐step approach produced 70 wt% liquid, mainly consisting of the lubricant fraction.	Mg_3_AlO_4.5_ trapped 30% of HCl at 280–300 °C under atmospheric pressure, increasing to ≈100% at 250 °C under an H_2_ pressure of 3 MPa.	Kots et al.^[^ ^112^ ^]^
16	2024	WEEE plastics (main polymers: ABS, HIPS, LDPE, PP, PCh), PAi), PMMAj), and EVAk))	Fe_2_CO_3_, CaO, ZSM‐5 (Si/Al = 45 mol. ratio), Fe/ZSM‐5, Ca/ZSM‐5, USYq) (Si/Al = 48 mol. ratio), Fe/USY, and Ca/USY	Two‐stage FxBR; pyrolysis: 575 °C; catalysis: 450 °C, feed ratio: 5 g h^−1^, total operation time: 4 h	Fe/ZSM‐5 was the best halogen removal, coke tolerance, and activity after regeneration. After regeneration, the oil yield decreased from 70.2 to 68.5 wt%, the aromatic hydrocarbon content decreased from 45.3 to 42.6 wt%, the Cl concentration decreased from 62 to 57 ppm, and the Br concentration increased from 119 to 123 ppm.	In catalytic pyrolysis, the Cl concentration decreased in the order: Fe_2_CO_3_ > ZSM‐5 > USY > CaO > Fe/USY > Ca/ZSM‐5 = Ca/USY > Fe/ZSM‐5(62 ppm). The Br concentration decreased in the order: ZSM‐5 > USY > Fe_2_CO_3_ > Ca/ZSM‐5 > Ca/USY > CaO > Fe/ZSM‐5 > Fe/USY (83 ppm).	López et al.^[^ ^120^ ^]^

a) Mixed plastic;

b) High‐impact polystyrene;

c) Printed circuit boards;

d) Waste;

e) Waste‐printed circuit boards;

f) Tetrabromobisphenol A;

g ^)^Acrylonitrile butadiene styrene;

h ^)^Polycarbonate;

i ^)^Polyamides;

j ^)^Polymethyl methacrylate;

k) Ethylen vinyl acetate;

l) Mobil crystalline material;

m) Red mud;

n) Silicoaluminophosphate;

o) Silica‐alumina;

p) Molar;

q) Ultrastable Y‐zeolite;

r) Fixed‐bed reactor;

s) Fluidized‐bed reactor;

t) Monoaromatic hydrocarbon;

u) Benzene, toluene, ethylbenzene, and xylene.

**Figure 7:**
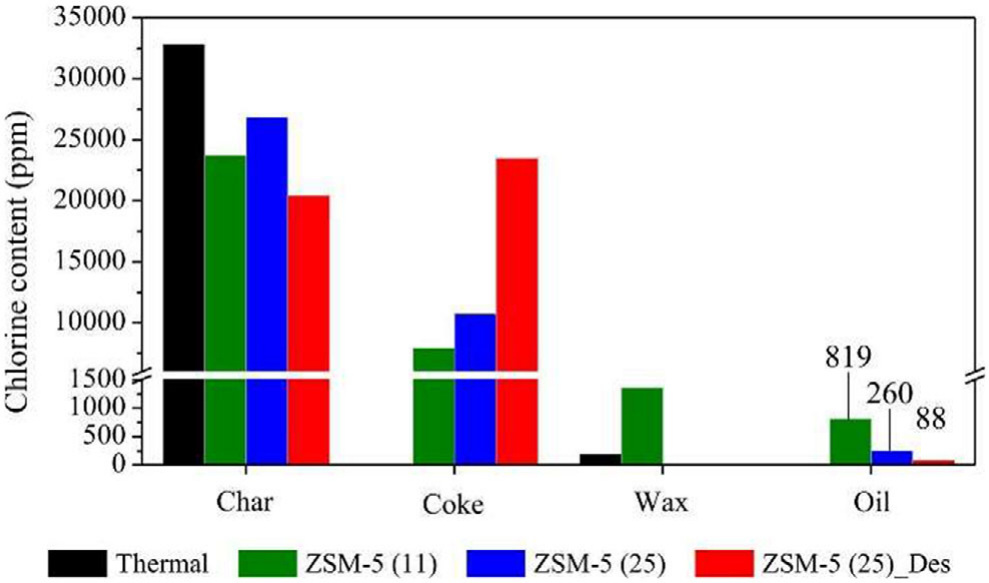
Thermal and catalytic pyrolysis of pretreated WEEE plastic: Cl concentration in various fractions. Reaction conditions: 600 ºC (thermal zone)/450 ºC (catalytic zone); The catalytst to feedstock ratio is 0.2. [Reproduced with permission.^[^ ^109^ ^]^ Copyright 2022, Elsevier (License number: 5955270641531)].

The ex situ mode allows for better optimization of pyrolysis conditions and catalyst configurations compared with the in situ mode. Bhaskar et al.^[^ ^107^ ^]^ (Table 4, entry 1) pyrolyzed a PE/PP/PS/PVC (weight ratio = 3/3/3/1) mixture using a Ca–C absorber (a calcium carbonate–carbon composite of 90 wt% calcined CaCO_3_ and 10 wt% phenolic resin) in a two‐stage fixed‐bed reactor. Across six batch experiments, oil and gas yields ranged from 65–75 to 15–23 wt%, respectively. Furthermore, in four of the six batch experiments, the Cl concentration was reduced to an undetectable level in the pyrolysis oil. Park et al.^[^ ^108^ ^]^ employed an auger reactor for thermal pretreatment in a two‐step pyrolysis process of mixed plastic waste (MPW), incorporating a hot filter with CaO to capture Cl from pyrolysis products (Table 4, entry 10). Thermal pretreatment at 300 °C reduced the Cl content in MPW from 8100 to 2800 ppm. Furthermore, CaO‐based Cl capture significantly reduced the Cl content in the pyrolysis oil to 68 ppm. The pyrolysis oil mainly consisted of MAHs (80 wt%), with benzene comprising a substantial 55 wt%. Marino et al.^[^ ^109^ ^]^ studied the pyrolysis of PVC‐containing WEEE plastics (Table 4, entry 11). A dechlorination rate of 87% was achieved by thermal pretreatment at 350 °C. The samples were subjected to catalytic pyrolysis in a two‐stage fixed‐bed reactor after thermal treatment. For pyrolysis alone, the wax yield was over 70 wt%, and the oil yield was less than 10 wt%. The oil yield increased to ≈60 wt% in the presence of desilicated ZSM‐5. The oil product of desilicated ZSM‐5 contained more than 50 wt% MAHs (mainly BTX). Furthermore, the Cl concentration in the oil decreased significantly, ranging from 260 to 819 ppm with ZSM‐5 to 88 ppm with desilicated ZSM‐5 (**Figure** **7** **)**.

**Figure 8:**
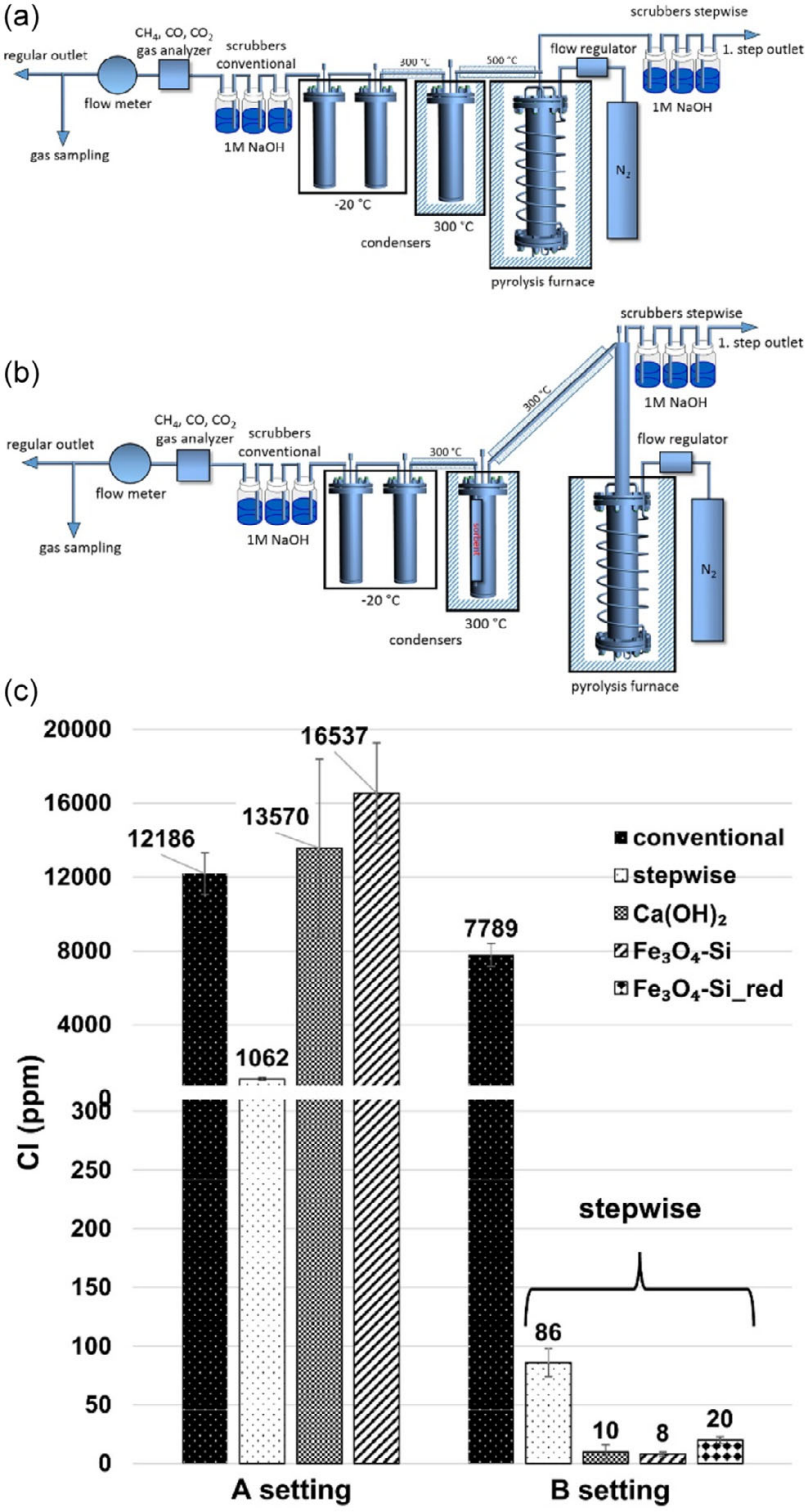
Pyrolysis unit settings a) without and b) with reflux extension. c) Cl content in liquid products. [Reproduced with permission.^[^ ^110^ ^]^ Copyright 2022, Elsevier (License number: 5955270991498)].

Hubacek et al.^[^ ^110^ ^]^ (Table 4, entry 13) investigated the stepwise pyrolysis of plastic mixtures using silica gel‐supported Fe_3_O_4_ catalysts (Fe_3_O_4_‐Si and Fe_3_O_4_‐Si_reduction). They studied the influence of pyrolysis unit settings (**Figure** **8**a,b) and the pyrolysis process on the dehalogenation of pyrolysis oil. In the configuration shown in Figure 8a, the absorption bed was constructed from steel mesh (in situ) and installed in the upper third of the reactor. In contrast, the configuration in Figure 8b featured an absorption bed made of steel tubing (ex situ), which was connected to an outlet of the first condenser running at 300 °C. In the pyrolysis process, conventional (500 °C) and stepwise pyrolysis (thermal pretreatment at 350 °C and pyrolysis at 500 °C) were considered. For conventional pyrolysis, the oil yield was 52.1 ± 1.6 wt% and the Cl concentration was 12,186 ppm for setting (a), and for setting (b), the oil yield was 63.3 ± 1.6 wt% and the Cl concentration was 7786 ppm. In setting (b), stepwise pyrolysis reduced the Cl concentration in the oil to 86 ppm, with an oil yield of 67.0 ± 0.7 wt%. Furthermore, the use of the catalysts reduced the Cl concentration from 3 to 23 ppm. Wang et al.^[^ ^111^ ^]^ (Table 4, entry 14) used carbon‐based catalysts (Fe/Ni/Mo@N/C) and HZSM‐5 for the pyrolysis of Cl‐containing mixed plastics (PE, PP, PS, and PVC). The results indicated that the combination of a carbon‐based catalyst and HZSM‐5 demonstrated higher selectivity (70.41%) for BTEX compared with that achieved using the conventional catalyst (MCM‐41 + HZSM‐5). In addition, they observed that the hydrothermal pretreatment of mixed plastics enhanced dechlorination and improved BTEX selectivity. Kots et al.^[^ ^112^ ^]^ (Table 4, entry 15) studied the catalytic pyrolysis of PVC and PP mixtures in an autoclave reactor using Mg_3_AlO_4.5_ and Ru/TiO_2_. They reported that Mg_3_AlO_4.5_ achieved quantitative dechlorination, while Ru/TiO_2_ converted the dechlorinated polymer into a 70 wt% oil, primarily consisting of lubricating oil, at 250 °C under a 3 MPa H_2_ atmosphere.

The behavior of Br during the pyrolysis of Br‐containing plastics has been studied in printed circuit boards (PCBs), WEEE, tetrabromobisphenol A (TBBPA), and high‐impact polystyrene (HIPS). Jung et al.^[^ ^113^ ^]^ (Table 4, entry 2) investigated the effects of CaO, Ca(OH)_2_, and oyster shells on Br removal during the pyrolysis of HIPS waste containing BFR and Sb_2_O_3_ in a fluidized‐bed reactor. The pyrolysis oil mainly consisted of styrene, ethylbenzene, cumene, and other aromatic hydrocarbons. During catalytic pyrolysis, the concentrations of ethylbenzene and cumene decreased, whereas the styrene concentration increased significantly. At 459 °C, the total Br content of the pyrolysis oil decreased from ≈5 to 1.3 and 2.7 wt% in the presence of Ca(OH)_2_ and oyster shells, respectively. Kumagai et al.^[^ ^114^ ^]^ (Table 4, entry 3) investigated the effects of Ca(OH)_2_ on the pyrolysis of both phenol‐ and epoxy‐resin paper‐laminated PCBs containing TBBPA. Pyrolysis experiments revealed a maximum removal of 94% HBr and 98% brominated phenol from the pyrolysis products. In addition, metal loss through the volatilization of brominated metals was substantially suppressed. Furthermore, Ca(OH)_2_ promoted the formation of phenolic compounds derived from the PCB matrix at temperatures below 300 °C, while the fixation of brominated compounds occurred at temperatures above 300 °C. Zhang et al.^[^ ^115^ ^]^ (Table 4, entry 8) performed microwave‐assisted pyrolysis of waste printed circuit boards (WPCB) to investigate the effects of K_2_CO_3_, Na_2_CO_3_, NaOH, ZSM‐5, and kaolin on the pyrolysis products and Br fixation. Adding K_2_CO_3_ significantly increased the concentration of phenol in the oil from 73.69% to 85.45%. The addition of ZSM‐5 and kaolin enhanced the concentration of monocyclic aromatic hydrocarbons (excluding phenol) in the oil, increasing it from 12.57% to 17.02% and 15.85%, respectively. In addition, the proportion of C_11–20_ compounds increased from 17.7% to 29.45% with ZSM‐5 and to 27.43% with kaolin. In addition, the Br fixation increased from 29.11% to 99.80%, 96.39%, and 86.69% with the addition of K_2_CO_3_, Na_2_CO_3_, and NaOH, respectively. Oleszek et al.^[^ ^116^ ^]^ (Table 4, entry 9) studied the distribution of pyrolysis products and Br compounds in TBBPA in the presence of Cu_2_O. In the presence of Cu_2_O, gas and oil production decreased, and char production increased. The pyrolysis oil primarily consisted of phenols, carbonyls, ethers, and aromatics. Notably, the addition of Cu_2_O significantly reduced the concentration of brominated organic compounds produced during the pyrolysis of TBBPA. The brominated organic content in the oil was reduced to 0.2 wt% at 480 °C and 0.9 wt% at 600 °C, while it was 6–7 wt% without Cu_2_O. Charitopoulou et al.^[^ ^117^ ^]^ (Table 4, entry 12) investigated the pyrolysis of Br‐containing plastic mixtures using ZSM‐5, Al_2_O_3_, MgO, Fe/Al_2_O_3_, and Fe/MgO. The catalysts promoted the formation of phenolic compounds, with Fe/Al_2_O_3_ demonstrating the highest selectivity. The debromination rate followed the order: Fe/Al_2_O_3_ > Fe/MgO > MgO > ZSM‐5 > Al_2_O_3_. Among the tested catalysts, Fe/Al_2_O_3_ was the most effective for both enhancing the formation of phenolic compounds and achieving debromination.

**Figure 9:**
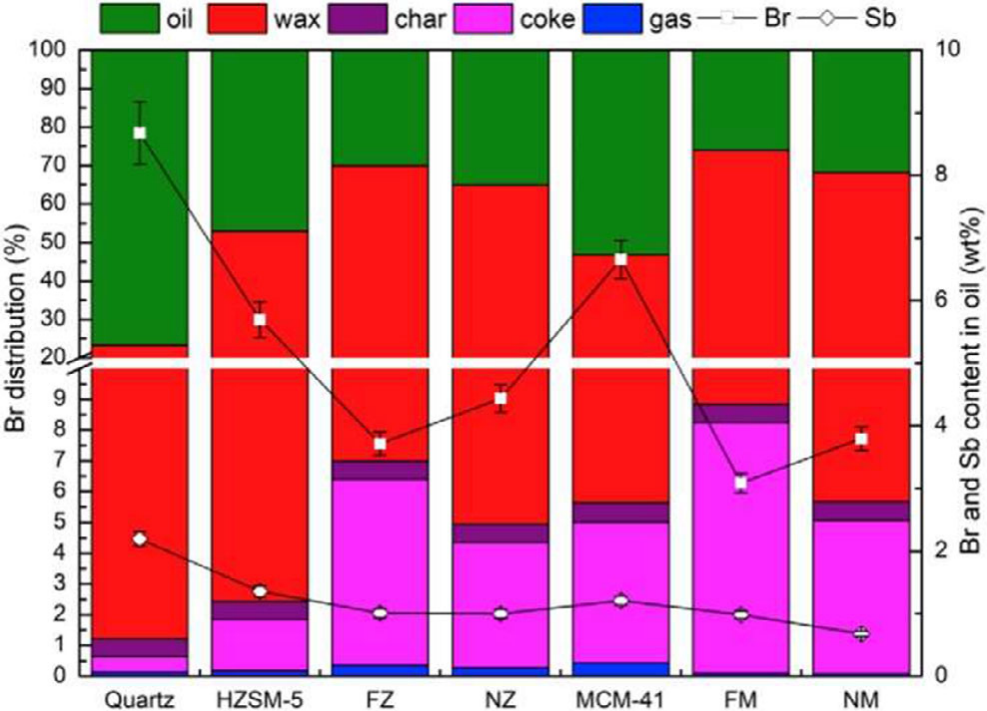
Br distribution and the Br and Sb content in oils from the pyrolysis–catalytic upgrading of Br‐HIPS using various catalysts. Error bars represent the standard deviation. [Reproduced with permission.^[^ ^118^ ^]^ Copyright 2017, Elsevier (License number: 5955271403113)].

**Figure 10:**
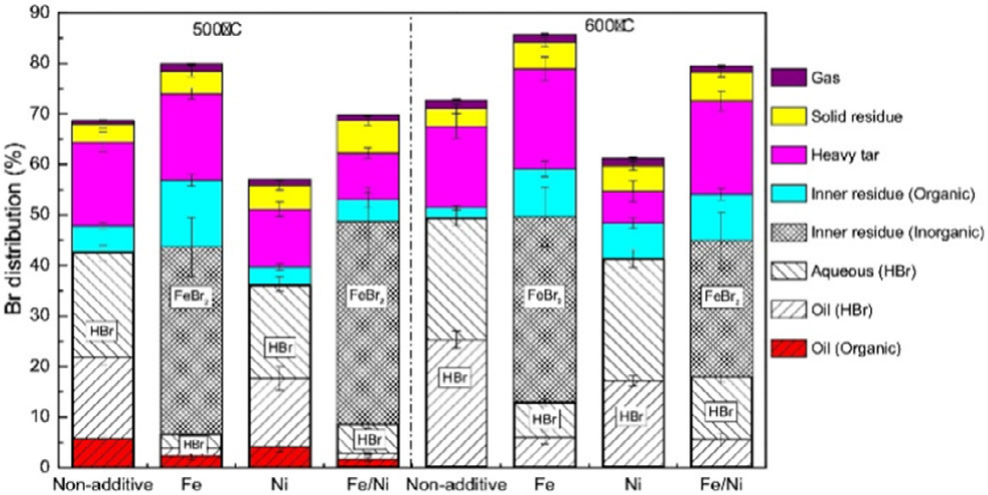
Br distribution in oils from PCB pyrolysis with Fe and Ni particles. [Reproduced with permission.^[^ ^119^ ^]^ Copyright 2018, Elsevier (License number: 5955280203997)].

**Figure 11:**
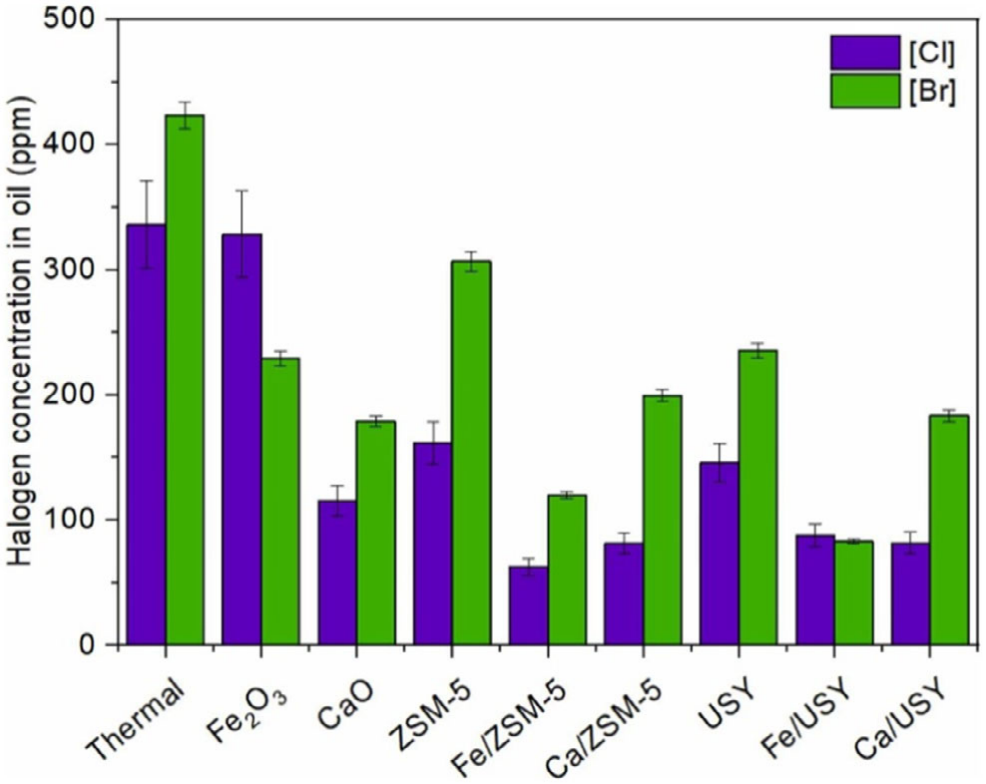
Overall Cl and Br concentrations in oils from the catalytic pyrolysis of WEEE‐derived plastics. [Reproduced with permission.^[^ ^120^ ^]^ Copyright 2024, Elsevier (License number: 5955280786654)].

Ma et al.^[^ ^118^ ^]^ studied the pyrolysis of HIPS using Fe‐ and Ni‐modified catalysts (Table 4, entry 4). Compared with oil obtained by pyrolysis (69.0 wt%), oil yields were 63.2 wt% for Fe/ZSM‐5, 61.2 wt% for Ni/ZSM‐5, 65.9 wt% for Fe/MCM‐41, and 65.3 wt% for Ni/MCM‐41. Regarding the oil composition, Fe‐modified catalysts increased the yield of monocyclic aromatics, whereas Ni‐modified catalysts increased the yield of bicyclic aromatics. Furthermore, the Fe‐modified catalyst effectively captured inorganic Br, favoring the removal of Br from the oil (**Figure** **9**). Ma and Kamo^[^ ^119^ ^]^ used Fe and Ni powders to pyrolyze PCBs in a two‐stage fixed‐bed reactor (Table 4, entry 5). The maximum oil yield (50.1 wt%) was obtained at 500 °C in the pyrolysis of PCBs. When Ni was added, the oil yield decreased to 29.1 wt%, and the gas yield increased to 9.6 wt% at 600 °C. Metal particles enhanced aromatic compound formation (phenol, benzene, and toluene) and achieved higher debromination ratios (Br content <2.9% in oil). Although the Br content in the oil was less than 2.9%, further removal of HBr was necessary to obtain Br‐free oil as chemical feedstock (**Figure** **10**). López et al.^[^ ^120^ ^]^ studied the catalytic pyrolysis of WEEE plastics in a two‐stage fixed‐bed reactor (Table 4, entry 16). WEEE plastics contained Cl (0.599 ± 0.239 wt%) and Br (0.225 ± 0.064 wt%), and the distribution behavior of the halogens was investigated using eight different catalysts (Fe_2_CO_3_, CaO, ZSM‐5, Fe/ZSM‐5, Ca/ZSM‐5, USY, Fe/USY, and Ca/USY). In the absence of the catalysts, the halogen concentrations in the oil were 336 and 423 ppm for Cl and Br, respectively. In catalytic pyrolysis, the Cl concentration in oil decreased in the order Fe_2_CO_3_ > ZSM‐5 > USY > CaO > Fe/USY > Ca/ZSM‐5 = Ca/USY > Fe/ZSM‐5 (minimum: 62 ppm), while the Br concentration decreased in the order ZSM‐5 > USY > Fe_2_CO_3_ > Ca/ZSM‐5 > Ca/USY > CaO > Fe/ZSM‐5 > Fe/USY (minimum: 83 ppm) (**Figure** **11**). In addition, Fe/ZSM‐5 and Fe/USY were used to evaluate the change in halogen concentration over time during the continuous pyrolysis of WEEE plastics. In Fe/USY, the halogen concentration increased with the operating time, reaching more than 120 ppm for both Cl and Br in 4 h. By contrast, for Fe/ZSM‐5, the halogen concentration in the oil remained constant regardless of the operating time, maintaining values of ≈60 and 120 ppm for Cl and Br, respectively. Coke deposition on the catalyst was lower for Fe/ZSM‐5 than for Fe/USY, indicating that Fe/ZSM‐5 has a superior halogen removal capacity and coke tolerance. The regeneration of the Fe/ZSM‐5 catalyst was also evaluated. After regeneration, compared with the fresh condition, the oil yield decreased slightly from 70.2 to 68.5 wt%, the aromatic hydrocarbon content decreased slightly from 45.3 to 42.6 wt%, and the Cl and Br concentrations increased slightly from 62.5 to 57.7 ppm and from 119.8 to 123.4 ppm, respectively.

Wet dehalogenation, which removes halogens from polymers through elimination or substitution reactions in a solvent, is a promising pretreatment technique for reducing halogen content. Subcritical water treatment has been investigated for this purpose. Xiu et al.^[^ ^121^ ^]^ performed subcritical water treatment of PVC and waste PCBs at temperatures ranging from 150 to 400 °C. For PVC, no significant dechlorination was observed below 200 °C, while the dechlorination rate reached 93% at 250 °C and nearly 100% at temperatures above 300 °C. Product analysis confirmed that chlorine from PVC was completely transferred to the aqueous phase. Similarly, for PCBs, the dechlorination rate began to increase at 250 °C, reaching ≈90% at 300 °C, and nearly 100% at temperatures above 350 °C. Furthermore, the effects of NaOH and ethanol addition on the subcritical water treatment of PVC containing diethylhexyl phthalate (DEHP) as a plasticizer were investigated.^[^ ^122^ ^]^ With NaOH addition, the dechlorination rate reached ≈90% at 250 ºC and nearly 100% at 350 ºC. In contrast, ethanol addition resulted in a lower dichlorination rate: 75% at 250 °C and ≈90% at 350 °C. These results indicate that both nucleophilic substitution and direct dehydrochlorination mechanisms were involved. Analysis of the oil products revealed that DEHP was decomposed through hydrolysis in NaOH and ester exchange in ethanol.

Another effective approach involves dehalogenation in ethylene glycol (EG)/NaOH solutions prior to pyrolysis.^[^ ^123^, ^124^, ^125^, ^126^, ^127^, ^128^ ^]^ Kameda et al.^[^ ^124^ ^]^ investigated the dechlorination of flexible and rigid PVC in a NaOH/EG solution using a ball mill. Higher dichlorination rates were achieved using spherical Y_2_O_3_‐ZrO_2_ grinding media. For flexible PVC, a 97% dichlorination rate was achieved at 190 °C after 2 h with 1 m NaOH/EG solution. For rigid PVC, the rate was 85% under similar conditions, but with a reaction time of 6 h. The authors concluded that grinding media enhanced the exposed surface area of PVC and improved contact with the solution. Dehalogenation of automobile shredder residue (ASR) using a NaOH/EG solution and ball milling was also reported.^[^ ^125^ ^]^ Treatment at 190 °C for 180 min in 0.5 M NaOH/EG solution resulted in 96% dechlorination and nearly 100% debromination. The chlorine and bromine contents in the treated ASR were reduced to less than 0.06 wt% and 0.01 wt%, respectively, making the material suitable for feedstock recycling. Moreover, an upscaled ball mill reactor was proposed for the dechlorination of PVC waste from actual end‐of‐life products.^[^ ^126^ ^]^ The cylindrical SUS304 reactors had dimensions of 26 cm in diameter and 60 cm in length. Waste samples included PVC sealing strips (SS) from refrigerators and PVC sheathing (CC) from electrical cables. In the case of SS, a 99% dechlorination rate was reached using 1.0 m NaOH/EG under various mechanical conditions. For CC, a 92% dichlorination rate was obtained at 0.5 m NaOH/EG with a ball size of 1.27 cm and a rotation speed of 45 rpm. Optimal conditions included high NaOH concentration, a large number of balls, and a high rotational speed for SS, whereas medium ball size and speed were suitable for CC. While these studies remain at laboratory scale, they demonstrated quantitative dehalogenation at 190 °C under atmospheric pressure.

### 4.2 PET Conversion into Chemical Feedstock by Pyrolysis

In PET pyrolysis, various catalysts have been investigated to suppress TPA formation and convert PET to oil, though the variety is less compared with polyolefins. Masuda et al.^[^ ^129^, ^130^ ^]^ studied PET pyrolysis using FeOOH, which effectively inhibited TPA formation and simultaneously converted it into acetophenone and benzene. Obuchi et al.^[^ ^131^ ^]^ pyrolyzed a mixture of PP and PET at 425 °C using a TiO_2_/SiO_2_ catalyst, resulting in ≈70% oil. The catalyst was regenerated at 400–500 °C in air. The fresh and regenerated catalysts were tested nine and seven times, respectively, and the oil yield was maintained at ≈70 wt% (catalyst: 5.0 g, PET/(PP + PET) = 0.15) during the repeated runs. Du et al.^[^ ^132^ ^]^ used ZSM‐5 for the catalytic pyrolysis of polyester carpets. Pyrolysis produces TPA‐ and benzoic acid‐rich oils.

By using ZSM‐5, benzene‐ and naphthalene‐rich oils were obtained. Diaz‐Silvarrey et al.^[^ ^133^ ^]^ studied the effects of sulfated zirconia on the pyrolysis of PET, which resulted in the enhanced decomposition of TPA into benzoic acid. Liu et al.^[^ ^134^ ^]^ performed PET pyrolysis in a fixed‐bed reactor using an activated carbon‐supported molybdenum oxide catalyst (MoO_2_/C(b‐MoO_2_/C)). At 400 °C, the highest yields of olefins and aromatic compounds were obtained, reaching 10.10 and 52.16 wt%, respectively. During pyrolysis under a nitrogen atmosphere, the concentrations of TPA and benzoic acid in the aromatic products reached 34.74% and 9.05%, respectively. In the presence of 20 wt% catalyst, their concentrations reached 28.64% and 25.07%, respectively.

**Figure 12:**
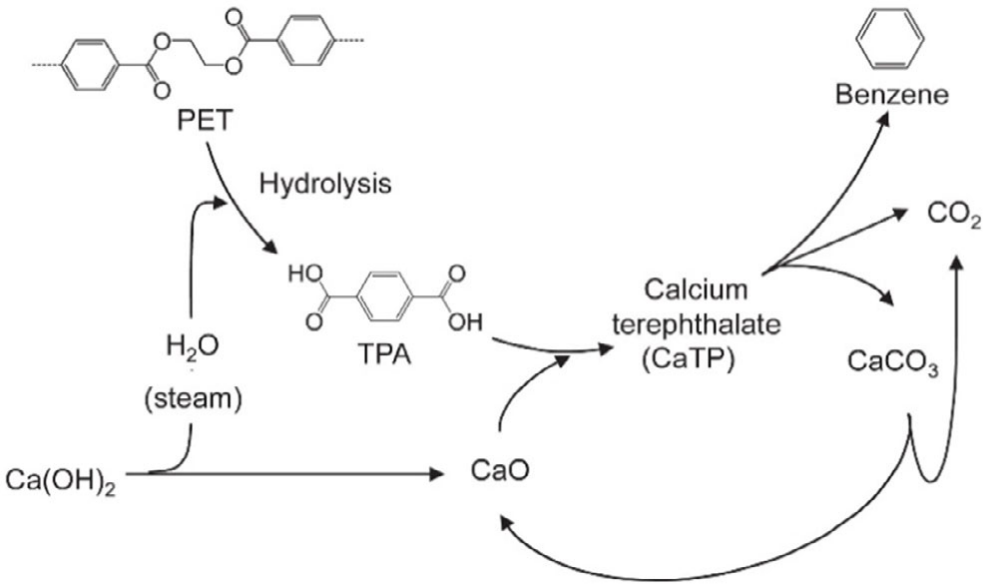
Reaction scheme for benzene production during the pyrolysis of a PET/Ca(OH)_2_ mixture. [Reproduced with permission.^[^ ^145^ ^]^ Copyright 2021, Oxford University Press (License number: 6031340143358)].

**Figure 13:**
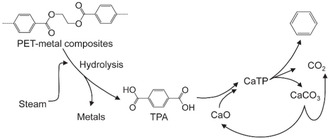
Reaction scheme for the two‐step process of benzene production from PET. [Reproduced with permission.^[^ ^145^ ^]^ Copyright 2021, Oxford University Press (License number: 6031340143358)].

**Figure 14:**
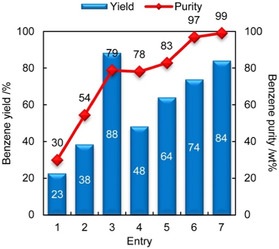
Benzene yield and purity in oil obtained under different processes and conditions. [Reproduced with permission.^[^ ^145^ ^]^ Copyright 2021, Oxford University Press (license number: 6031340143358)].

The authors investigated a process for converting TPA into benzene, which is an intermediate petrochemical feedstock, using Ca(OH)_2_ and CaO as reactants, which are inexpensive and less resource competitive.^[^ ^135^, ^136^, ^137^, ^138^, ^139^, ^140^ ^]^ A one‐step process for the pyrolysis of a PET/Ca(OH)_2_ mixture^[^ ^135^, ^137^ ^]^ (**Figure** **12**) and two‐step process for the hydrolysis of PET by steam and subsequent decarboxylation of vaporized TPA by CaO^[^ ^136^, ^138^, ^139^, ^140^ ^]^ (**Figure** **13**) have been developed. In the simple pyrolysis of PET (**Table** **5**, entry 1; **Figure** **14**), the benzene yield and purity were only 23% and 30 wt%, respectively. The pyrolysis of a mixture of PET:Ca(OH)_2_ (1:10 molar ratio) (Table 5, entry 3; Figure 14) significantly improved the benzene yield (88%) and purity (79 wt%) due to the hydrolysis of PET by steam from Ca(OH)_2_ dehydration and subsequent decarboxylation of TPA by CaO (Figure 12). In addition, high benzene yields were obtained because Ca(OH)_2_ and PET were pyrolyzed in a mixed state. However, the reaction requires relatively high temperatures, which leads to side reactions occurring and a reduced benzene purity. The benzene yield obtained in the two‐step process is lower than that obtained in the one‐step process, whereas the benzene purity is higher. In particular, the benzene yield and purity reached 74% and 97 wt%, respectively, by controlling the steam decomposition temperature of PET (Table 5, entry 6; Figure 14).^[^ ^136^ ^]^ The reaction with CaO is based on a pyrolysis reaction, and other plastics, such as PE and PP, can be simultaneously pyrolyzed with PET.^[^ ^138^ ^]^

**Table 5:** Experimental conditions for PET conversion to benzene.

Entrya)	Sample	Process	Bed material	Hydrolysis temperature [°C]	Bed temperature [°C]	References
1	PET	One step	–	–	700	[135]
2	PET	One step	CaO	–	700	[135]
3	PET	One step	Ca(OH)_2_	–	700	[137]
4	PET	Two step	CaO	450	700	[137]
5	PET	Two step	CaO	400	500	[136]
6	PET	Two step	CaO	300–500 (2 °C min^−1^)	500	[136]
7	Terephthalic acid	Two step	CaO	450	600	[139]

a) The results are presented in Figure 9.

## 5 Summary and Outlook

As discussed in Section 2, numerous publications have comprehensively reported the impacts of plastic, catalyst, and reactor types to optimize oil recovery with higher yields, purity, and added value as chemical feedstocks and fuels. SA and mesoporous silica maintained a comparatively high oil yield from polyolefins and narrowed the carbon number distribution of the obtained oil. Therefore, these catalysts are beneficial for recovering aliphatic hydrocarbons from PE‐ and PP‐based plastics. Zeolite catalysts, which are more acidic than SA and mesoporous silica, enhance the production of shorter‐chain hydrocarbons and aromatic hydrocarbons, such as BTX. Metal‐loaded zeolites, such as Ga‐loaded zeolites, further enhance BTX production and improve catalyst durability. Thus, zeolite‐based catalysts can increase the production of aromatics from plastic waste. Sulfated zirconia, metal oxides (such as MgO and ZnO), and metal‐supported catalysts (Pt/Al_2_O_3_, Pt/SiO_2_) have also been used. In particular, ZnO is highly stable and has proven effective in producing oil equivalent to lubricating oil. Although other catalysts have been investigated, the product selectivity mainly depends on the acidity and reaction temperature of the catalysts. Thus, it can be concluded that the current lab‐scale approach for polyolefins allows for the control and improvement of the product yield and selectivity at a comparatively high technical level.

The investigation of the industrial‐scale pyrolytic liquefaction projects in Section 3 revealed that pyrolytic liquefaction plants with a plastic processing capacity of tens of thousands have been developed and operated for mainly treating common plastics such as PE, PP, and PS. Most plants operate without a catalyst, and only a few have commercialized the catalytic process. This indicates that catalytic approaches on the industrial scale still face many hurdles. This investigation confirmed that catalyst durability and reusability are crucial for achieving stable, long‐term operation and reducing the operational costs of catalytic processes. Thus, the development of highly durable catalysts and optimization of the process design are required to maximize the benefits of several catalysts for the recovery of high‐value products.

Another vital step in enhancing the recycling rate of waste plastics is to expand the types of treatable plastics. Therefore, Section 4 investigated the current progress in treating halogen‐containing plastics and PET, which are hard‐to‐recycle plastics in pyrolysis. According to a comprehensive literature survey, over 90% halogen removal from the pyrolysis products has been achieved in laboratory‐scale studies, and the halogen concentration in the oil has been reduced to less than 100 ppm. Alkaline metals and alkaline earth metal oxides are inexpensive and effective halogen absorbents and further investigations of their regeneration after halogen removal are required for their application in industrial processes. Fe‐impregnated catalysts showed excellent performance in terms of simultaneous dehalogenation, product yield, and selectivity. However, further developments, including improved durability and recyclability, are required for industrial applications. Wet dehalogenation is another key method for reducing halogen content in waste plastics before pyrolysis, where halogens are removed from the polymer through elimination or substitution reactions in an EG/NaOH solution.^[^ ^121^, ^122^, ^123^, ^124^, ^125^, ^126^, ^127^, ^128^ ^]^ The problem with PET pyrolysis is the production of TPA, which has a high boiling point and acidic compounds. The effectiveness of Ca(OH)_2_ and CaO, as inexpensive materials, in decomposing PET into benzene has been reported, although it requires comparatively high temperatures above 500 °C. Therefore, the development of effective and durable decarboxylation catalysts that operate at lower temperatures is essential.

Although plastic recycling is ongoing, many processes remain outside the carbon cycle loop. Pyrolysis offers the potential to handle waste plastics with diverse compositions and qualities, presenting a significant advantage over mechanical recycling and monomer recovery methods. However, due to the wide variety of products generated from pyrolysis, collaboration with the petroleum and petrochemical industries is indispensable for their full and effective utilization. Japan has 20 refineries with a combined crude oil processing capacity of 3,230,400 bbl d^−1^ as of the end of Oct 2023.^[^ ^141^ ^]^ By contrast, Japan treated 145,23 gigaliter (GL) of crude oil in 2020, with 13.38 GL (9%) of naphtha refined and 26.37 GL of naphtha imported, totaling 39.74 GL. That same year, the volume of waste plastics amounted to 8220 kt, which was ≈3% of the total imported crude oil and naphtha.^[^ ^142^ ^]^ Thus, the amount of plastic waste is small compared with the amount of crude oil consumed, presenting significant advantages in utilizing existing oil refining facilities for processing waste plastics.

From a circular economy perspective, one of the greatest challenges in chemical recycling remains its cost. However, increasing demand for recycled plastics, driven in part by EU's mandatory recycled content requirements, is expected to stimulate scale‐up and reduce costs to more competitive levels. In practical terms, matching the quality of collected plastics with the appropriate recycling technologies is critical. Expanding the range of acceptable plastic feedstocks and improving recovery rates are key technological objectives. Moreover, pretreatment is crucial to ensure that recycled materials meet the quality standards necessary for chemical recycling. It is essential to exclude plastics incompatible with chemical recycling processes—such as PVC, BFR‐containing plastics, PET—from collected waste streams. In practice, many packaging plastics consist of multilayer structures combining different polymers and may also include aluminum or paper. Plastics in automobiles and electrical appliances are often combined with metals in complex assemblies, which complicates separation. In addition to conventional physical separation methods (e.g., based on specific gravity), optical sensors have recently become widespread for material identification and sorting. Furthermore, methods such as digital product passports and digital watermarks have been developed to encode information on material composition and recyclability directly onto products.^[^ ^143^, ^144^ ^]^ These methods will be crucial for aligning the supply of suitable recycled plastics with the demands of chemical recycling facilities. As interest in chemical recycling—including pyrolysis—continues to grow both in Japan and internationally, we hope that the insights presented in this review will contribute to the advancement of a circular economy and the achievement of carbon neutrality.

## Conflict of Interest

The authors declare no conflict of interest.
